# Meta-analysis: A useful tool to assess infection prevalence and disease ecology of *Borrelia burgdorferi* sensu lato in nymphal ticks in North-Western Europe with recommendations for a standardised approach to future studies

**DOI:** 10.1016/j.parepi.2022.e00254

**Published:** 2022-05-31

**Authors:** R. Walsh, M. Gormally, A. Zintl, C. Carlin

**Affiliations:** a University of Galway, Applied Ecology Unit, Earth and Life Sciences, School of Natural Sciences and Ryan Institute, University of Galway, University Road, Galway, Ireland; b University College Dublin, Applied Ecology Unit, Earth and Life Sciences, School of Natural Sciences and Ryan Institute, University of Galway, University Road, Galway, Ireland

**Keywords:** Lyme borreliosis, meta-analysis of proportions, Disease ecology, *Ixodes ricinus*

## Abstract

Lyme borreliosis is a vector-borne disease of concern in Europe. While neuroborreliosis data are reportable at EU level, it can nevertheless be difficult to make comparisons of disease risk between neighbouring countries. This study used proportion meta-analyses to compare environmental markers of disease risk between woodland sites in two countries in North-Western Europe (Ireland, Scotland). 73 site-visits from 12 publications were analysed, resulting in a significantly higher pooled nymphal infection prevalence (NIP) in Ireland (8.2% (95% CI: 5.9–11.4%)) than Scotland (1.7%(95% CI 1.1–2.5%)). All other analysed parameters of disease risk were also higher in Ireland than Scotland. Subgroup-meta-analyses and meta-regressions were used to assess the influence of environmental variables on NIP. NIP increased significantly with increasing woodland size in Ireland, but not Scotland, which may be accounted for by Ireland's highly fragmented landscape.

Assuming the application of strict inclusion/exclusion criteria and control of variables, proportion meta-analysis can provide useful insights in disease ecology, as it allows for the achievement of high study powers incorporating samples collected across multiple sites, which is otherwise often a prohibitively difficult and resource-heavy feat in environmental studies in disease ecology. A standardised approach to data collection is recommended to achieve more robust meta-analyses in future in conjunction with additional research on environmental factors affecting Lyme borreliosis risk in Europe, particularly pertaining to the impact of host species on NIP.

## 1 Introduction

Lyme borreliosis is caused by bacteria of the *Borrelia burgdorferi* sensu lato (s.l.) complex (Spirochaetales: Spirochaetacea*,* Johnson et al., 1984 emend. Baranton et al., 1992). Its most common vector is the ectoparasite *I. ricinus* (Ixodida: Ixodidae, Linnaeus 1758) (Zintl et al., 2017), which parasitises a wide range of mammalian, avian, and reptilian hosts. In addition to *B. burgdorferi*, *I. ricinus* also serves as a vector for several other zoonotic tick-borne pathogens, including Babesia spp., a number of Rickettsiae, and Tick-Borne Encephalitis virus (Zintl et al., 2017). Lyme disease is the most common tick-borne illness in Europe (Kilpatrick et al., 2017; Cairns et al., 2019; Vandekerckhove et al., 2021). While the incidence of Lyme borreliosis varies throughout Europe (Vandekerckhove et al., 2021; Lindgren and Jaenson, 2006), the overall estimated population-weighted incidence of the disease in Western Europe alone has been placed at 22.04/100,000 (Sykes and Makiello, 2017). Symptoms in humans range from a rash and general malaise, to meningitis and arthritis (Cullen, 2010). The surveillance of Lyme borreliosis is crucial to the understanding, control, and diagnosis of this illness (van den Wijngaard et al., 2017). A range of surveillance methods are currently used to monitor the epidemiology of Lyme borreliosis and to give a sense of the overall risk of the disease in an area (van den Wijngaard et al., 2017). These methods can be split into: a) human/patient-centric methods which include the reporting of erythema migrans, sero-positive cases, and signs of disseminated infection such as neuroborreliosis (involvement of the central nervous system) (van den Wijngaard et al., 2017); and b) environmental survey methods (van den Wijngaard et al., 2017) which include surveys of infected ticks, and infected wildlife (van den Wijngaard et al., 2017).

### 1.1 Human/patient-centric methods

Due to differences in data collection methods, and an inherent difficulty in the diagnosis of Lyme borreliosis in humans, it can often be difficult to compare disease risk between different countries, even neighbouring jurisdictions, using human epidemiological data alone. In Ireland, a HPSC (Health Protection Surveillance Centre) report estimated the overall incidence of Lyme borreliosis at approximately 2–200 cases per annum (which approximates 0.2 to 4 per 100,000 population per annum), while noting that figures based on notified case rates usually underestimate overall rates of disease (Cairns et al., 2019; McKeown and Jackson, 2018). In neighbouring Scotland, however, 308 cases of Lyme borreliosis (PHS, 2019) were reported in 2019 which approximates an incidence of 5.64 per 100,000 population. On the other hand, a separate study of primary care data, which takes into account clinical diagnoses in addition to seropositivity, has put the incidence of diagnosed Lyme borreliosis in Scotland at 37.3 per 100,000 persons per year suggesting that this disease may often be underreported in the UK (Cairns et al., 2019).

A more standardised way of comparing disease risk between countries using human-centric data is to compare incidences of neuroborreliosis, which is reportable at European level since 2019 (HPSC, 2019). As there are set criteria for the diagnosis of neuroborreliosis, it should be possible to make a standardised comparison between countries where data on neuroborreliosis rates are available. In the case of Ireland the reported incidence of neuroborreliosis (2016–2020) ranged from 0.1–0.4 per 100,000 population per annum (HPSC, 2021). However, in neighbouring Scotland, neuroborreliosis data was not reported (PHS, 2019). In addition, there is a lack of consistency on how rates of diagnoses of neuroborreliosis translate to overall Lyme borreliosis disease burden with neuroborreliosis being cited as occurring in 3–38% of all diagnosed cases of Lyme borreliosis in Europe (van den Wijngaard et al., 2017), or in up to 12% of cases in Europe (HPSC, 2019) depending on the publication being referenced. The percentage of cases manifesting with neuroborreliosis is chiefly determined by the strains of *B. burgdorferi* s.l. present in a given country or region, with *Borrelia garinii* being the strain most associated with neurological symptoms (Zintl et al., 2017).

### 1.2 Environmental survey methods

Environmental survey methods used in European studies to assess the risk of Lyme Borreliosis include the use of nymphal tick infection prevalence (NIP) and density of infected nymphs (DIN) as markers of disease risk to humans (LoGiudice et al., 2008). As not all researchers collect tick density data in the field (LoGiudice et al., 2008; LoGiudice et al., 2003; Ostfeld and Keesing, 2000; Strnad et al., 2017; Gilbert, 2016), NIP is commonly used. Ticks collected across a number of sites in a region or country can be tested for the presence of *B. burgdorferi* s.l., and an assessment on NIP and DIN for the region can be calculated from this data. However, the resources and people-power to collect such data from multiple sites across a region or country can limit the universal applicability of this method (van den Wijngaard et al., 2017).

Where the collection of environmental data on Lyme borreliosis risk can be achieved, such studies can provide useful insights into the ecological factors affecting the ecology of the disease (van den Wijngaard et al., 2017), as well as supplementing disease risk data. NIP and DIN are influenced by various environmental factors, including climate (Zintl et al., 2017; Hvidsten et al., 2015), habitat type (Gilbert, 2016; Pfäffle et al., 2013; Estrada-Peña et al., 2015), habitat fragmentation (Kilpatrick et al., 2017; LoGiudice et al., 2008; Gilbert, 2016; Pfäffle et al., 2013), and the vertebrate host community (LoGiudice et al., 2008; Ostfeld and Keesing, 2000; Pfäffle et al., 2013; Keesing et al., 2010). Studies from North America, for example, have found that smaller woodland fragments are associated with higher tick densities (Ostfeld and Keesing, 2000), and that woodland size is inversely correlated with Lyme borreliosis risk (Ostfeld and Keesing, 2000; Pfäffle et al., 2013; Allan et al., 2003). North American data also highlights that that the vertebrate host community and host species diversity in an area has a major influence on NIP (LoGiudice et al., 2008; Ostfeld and Keesing, 2000; Keesing et al., 2010; Halsey and Miller, 2020). However, It is important to note that the disease ecosystem in North America involves different primary strains of *B. burgdorferi* s.l., with *B. burgdorferi* sensu stricto (s.s.) largely dominating the North American disease system, while the burden of disease in Europe is chiefly due to five pathogenic strains – *Borrelia afzelii, B. burgdorferi* s.s*., B. garinii, Borrelia bavariensis,* and *Borrelia spielmanii* (Kilpatrick et al., 2017). The North American system also involves different vector species and different wildlife host species and therefore differs considerably from that of north-western Europe where the relationship between woodland size (Gilbert, 2016), vertebrate host species (Mysterud et al., 2016), and disease risk is less clear cut. Even within the European system, Ireland represents a slightly unusual area in that it is particularly host-species poor (Baquero and Telleria, 2001) and highly fragmented (Copernicus, 2020).

Therefore, more directed studies regarding the influence of vertebrate host communities on NIP and DIN in the north-western European disease system are required (Gilbert, 2016; Millins et al., 2016).

While it can be difficult to gather enough data in primary studies to answer the broader questions on how environmental factors affect disease risk, as the study power is often dependent on sample catch, the use of a meta-analytic technique allows for the combination of several smaller studies to summarise an overall effect. Most meta-analyses are used to determine metrics of differences in effect size between groups (e.g. odds ratio, relative risk) (Wang, 2018). Conversely, a meta-analysis of proportions is a one-armed technique seeking to synthesise a single highly accurate measure of proportion (e.g. infection prevalence) from several smaller studies. This is achieved via the synthesis of a weighted average proportion – the average of the outcome of multiple studies, weighted by the inverse of the studies' sample variances (Wang, 2018). Furthermore, sub-grouping or regression analysis (‘meta-regression’) can be applied to investigate the effects of categorical or continuous variables respectively on the weighted average proportion.

Hence, a proportion-based meta-analysis in which markers of infection prevalence in a disease vector are summarised and sub-grouped by country has the potential to allow for the statistically robust comparison of risk markers of vector-borne diseases such as Lyme borreliosis between countries. This can, in turn, provide useful data to aid in disease surveillance decision making.

Given the somewhat surprising differences in human-centric estimates of disease incidence between Ireland and Scotland, it was decided (using published data from each country) to test whether a meta-analytical approach might be a useful tool for comparative purposes. Both countries are geographically similar with an oceanic climate and they both represent the North-Western European *B. burgdorferi* s.l. disease system.

This study uses a structured literature review and meta-analysis of proportions with sub-group analysis to compare environmental markers of disease risk (with NIP as the primary marker, and DIN as a secondary marker) between Scotland and Ireland, thus creating a risk-comparison based on standardised data, which can be added to the data generated by epidemiological reports and serological studies. The study also uses the technique of sub-group meta-analysis to determine the impact habitat factors described in the literature have upon disease risk in a geographically isolated and host-species poor region of Europe. Finally, the study appraises the use of meta-analytic techniques in the field of disease ecology with a view to making recommendations for streamlining reporting methods used in the literature for the benefit of future meta-analyses.

## 2 Material and methods

### 2.1 Search strategy

**Fig. 1:**
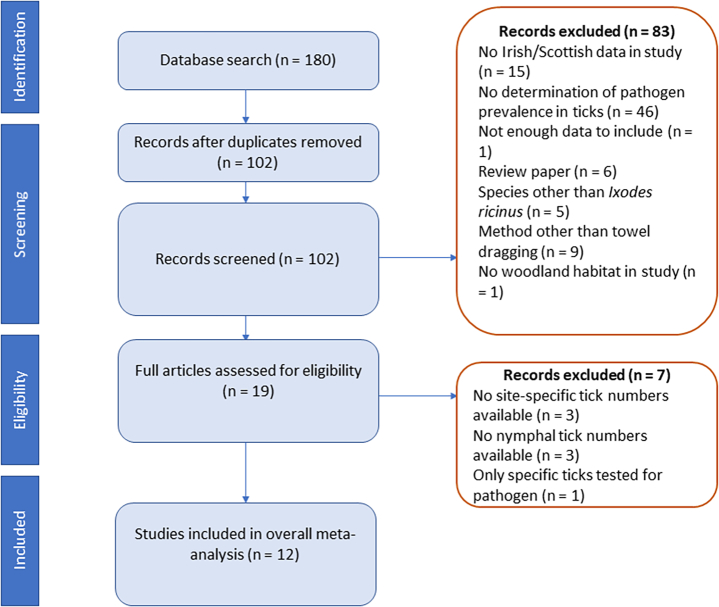
PRISMA flow diagram for the selection of studies for inclusion in the meta-analyses.

Three databases, PubMed, Scopus, and BIOSIS were searched on 04/03/2020 and 28/04/2020. The search string used contained the terms ‘tick’, ‘*Borrelia*’, and ‘Ireland’ or ‘Scotland’ or ‘UK’, with no year cut-off. After removal of duplicates, 102 results were returned (Fig. 1), all English-language results. The titles, and then abstracts and full papers of these were analysed for relevance to the meta-analysis.

### 2.2 Paper eligibility

Studies which used blanket-dragging or flagging to collect nymphal ticks in Scotland or Ireland, and in which tick samples were tested for *B. burgdorferi* s.l*.* were considered eligible for inclusion. Studies which used methodologies other than blanket-dragging, or in which the number of infected ticks was not disclosed were excluded. To reduce sample variability, only studies which sampled ticks in woodland habitats (the preferred habitat of ticks in Europe (ECDC, 2014)) on the Irish/Scottish mainland were included. Nineteen studies (Zintl et al., 2017; Millins et al., 2016; Lambert et al., 2019; Pichon et al., 2003; Pichon et al., 2005; Gray et al., 2000; Gray et al., 1999; Kirstein et al., 1997a; Kirstein et al., 1997b; Gray et al., 1995; Gray et al., 1992; Gray et al., 1996; Curtin and Pennington, 1994; Davidson et al., 1999; James et al., 2014; James et al., 2013; Ling et al., 2000; Millins et al., 2018; Bettridge et al., 2013) met these criteria (Table1).

### 2.3 Individual sites within papers

Most papers included data from several different study sites, each of which had varying properties (different woodland types, sizes, hosts present etc). As the authors of this paper were interested in establishing the effects of site-related variables on NIP and DIN, it was necessary to extract data from papers on a site-by-site basis. Therefore, some papers which did not provide site-specific data on nymph numbers and infection rates were excluded at this stage (Fig. 1). For papers that did provide site-specific data, a further round of eligibility criteria was applied, this time at individual site level, rather than at paper level. Information from each individual site visit, where reported, was collected separately. The site-specific eligibility criteria were as follows:

Woodland site on mainland Ireland/Scotland.

Total nymphal ticks collected and number/percentage positive for *B. burgdorferi* s.l. reported.

No conditions (e.g. positive blood meal analysis) attached to testing of ticks for pathogen.

Following the application of these criteria, there remained 74 eligible site visits from 12 papers (Table 1).

**Table 1:** Characteristics of the studies and sites meeting inclusion criteria for meta-analyses investigating pooled NIP and DIN.

	Author/Publication Year	Country	Number of sites	Information on individual sites?	No. sites meeting inclusion criteria	No. sites visits meeting inclusion criteria	Data from this paper included in meta-analysis?
	Lambert et al., 2019 (Lambert et al., 2019)	Ireland	8	Yes	0	0	No
	Zintl et al., 2017 (Zintl et al., 2017)	Ireland	13	No	0	0	No
	Pichon et al., 2005 (Pichon et al., 2005)	Ireland	3	Yes	2	4	Yes
	Pichon et al., 2003 (Pichon et al., 2003)	Ireland	1	Yes	0	0	No
	Gray et al., 2000 (Gray et al., 2000)	Ireland	1	Yes	1	1	Yes
	Gray et al., 1999 (Gray et al., 1999)	Ireland	8	Yes	7	3	Yes
	Kirstein et al., 1997b (Kirstein et al., 1997b)	Ireland	6	Yes	5	5	Yes
	Kirstein et al., 1997a (Kirstein et al., 1997a)	Ireland	5	Yes	3	3	Yes
	Gray et al., 1996 (Gray et al., 1996)	Ireland	1	Yes	1	1	Yes
	Gray et al., 1995 (Gray et al., 1995)	Ireland	24	Yes	7	7	Yes
	Gray et al., 1992 (Gray et al., 1992)	Ireland	2	Yes	2	6	Yes
	Bettridge et al., 2013 (Bettridge et al., 2013)	Scotland	17	Yes	1	1	Yes
	Curtin et al., 1994 (Curtin and Pennington, 1994)	Scotland	2	Yes	1	1	Yes
	Davidson et al., 1999 (Davidson et al., 1999)	Scotland	3	Yes	0	0	No
	James et al., 2013 (James et al., 2013)	Scotland	25	No	0	0	No
	James et al., 2014 (James et al., 2014)	Scotland	25	No	0	0	No
	Ling et al., 2000 (Ling et al., 2000)	Scotland	2	Yes	0	0	No
	Millins et al., 2018 (Millins et al., 2018)	Scotland	18	Yes	6	11	Yes
	Millins et al., 2016 (Millins et al., 2016)	Scotland	25	Yes	24	30	Yes
Total	19	2	189	16	61	74	12

### 2.4 Data extracted

All papers were closely scrutinised as an initial step. Data on the following were extracted from each paper for each site:

Number and (where provided) density of nymphal ticks collected.

Number/percentage of nymphal ticks positive for *B. burgdorferi* s.l. and (where provided) number/percentage of nymphal ticks positive for specific strains of *B. burgdorferi* s.l.

Each paper was then combed for data on habitat-related variables known to impact NIP and DIN, i.e. habitat type, habitat size, and hosts present.

#### 2.4.1 Habitat type

As ‘woodland site’ was an eligibility criterion, data on woodland type were available for all included sites. Woodland type was categorised as ‘deciduous’; ‘coniferous’; or ‘mixed deciduous and coniferous’ for the purposes of this analysis. This categorisation was based on what was reported by study authors.

#### 2.4.2 Habitat size

To ensure that woodland sizes were compared in a standardised way, Corine landcover data (100 m resolution) (Copernicus, 2020) from the 2000 or 2018 maps (based on the year of study publication) were used to define the size of the continuous woodland stand surrounding the study site. This was only possible where either the site name or geographic location was available. Where this information was not included for a site, it was excluded from the size meta-regression analysis.

#### 2.4.3 Hosts

Data on the presence or abundance of vertebrate host species (aside from deer) were rarely reported by authors, and when included, were collected in a highly variable way. Thus, this parameter was excluded from the meta-analysis, except for ‘deer’ (not deer species specific) as most studies contained some reference to the presence/exclusion/ability to record deer at specific sites. Some studies made reference to deer ‘herd size’ or to abundance estimated from faecal transects. To standardise across studies, deer data were only extracted where a herd size was mentioned by authors or where deer density was specifically included. Where herd size was measured, density was calculated by placing this number against the size of the woodland. A deer density of ‘0’ was only included where deer were specifically studied and deemed absent, or where the site in question was expressly within a deer exclusion fence.

#### 2.4.4 Assessment of study location and targeted sample collection

**Fig. 2:**
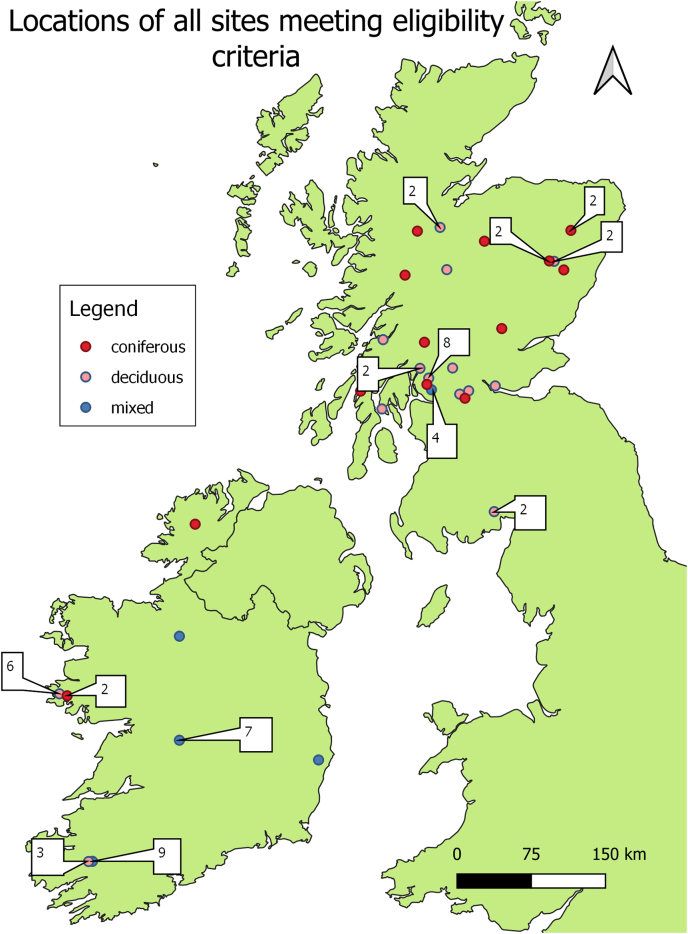
Locations of all sites included in analyses, with numbers indicating where several sites or site-visits occurred in one geographic area.

All available site locations included in the meta-analysis were mapped using QGIS 3.14 (QGIS.org, 2022) (Fig. 2). There was a wide geographical spread of sites from Scotland included in the analysis. Of the Irish sites the majority were located in the west of the island (Portumna, Connemara, Killarney). To assess the possible over-representation of certain regions in Ireland on NIP, a meta-analysis with subgrouping by site location was performed. There was no significant difference in overall NIP when broken down by location (Appendix A).

#### 2.4.5 Data availability

The number of papers with the data needed for inclusion in each analysis is listed in Table 2. If only two or fewer papers had the data needed for a meta-analysis, that analysis was not performed to avoid simply duplicating the findings of the papers' authors.

**Table 2:** Number of papers which met inclusion criteria and had sufficient data available to be included for each meta-analysis.

	Ireland analysis	Scotland analysis	Ireland and Scotland analysis
Overall NIP	8 Papers	4 Papers	12 papers
Overall DIN	3 papers	2 papers	5 papers
Overall Strain-specific NIP	4 papers	3 papers	7 papers
Woodland Type vs NIP	8 papers	4 papers	
Woodland Type vs strain - specific NIP	3 papers	2 papers⁎	
Woodland Type vs DIN	3 papers	2 papers⁎	
Woodland Size vs NIP	9 papers	3 papers	
Woodland Size vs Strain-specific NIP	8 papers	3 papers	
Woodland size vs DIN	3 papers	2 papers⁎	
Deer abundance vs NIP	4 papers	2 papers⁎	
Deer abundance vs Strain-specific NIP	3 papers	2 papers⁎	
Deer abundance vs DIN	2 papers⁎	2 papers⁎	

⁎ meta-analysis not done.

#### 2.4.6 Meta-analysis

All meta-analyses were performed using the metafor (Viechtbauer, 2010) package in R (R Core Team, 2019). Where possible, a GLMM meta-analysis was performed. An inverse meta-analysis was performed in the few cases where a GLMM model could not be fitted. The I^2^ statistic was used to interpret the heterogeneity of the data used in each meta-analysis. This statistic refers to variability in the data between studies. It is often high (e.g. 90%) in a proportion-based meta-analysis (Borenstein, 2009). As is convention, heterogeneity was defined as high (>75%), medium (>50%) or low (<50%) (Ahaduzzaman, 2019). A meta-regression was performed in the setting of a continuous explanatory variable (e.g. site size).

As is convention, the results of meta-analyses in this study are displayed as forest plots, displaying the confidence intervals and outcomes of each individual study, as well as the pooled proportion and confidence intervals for the overall effect.

The results of meta-regressions are displayed as bubble plots displaying the size of each individual study as well as a regression line for the relationship between explanatory and dependent variables.

#### 2.4.7 Publication bias

The risk of publication bias for all studies was considered to be low. As observational (i.e. non-comparative) studies, the prevalence outcomes of papers included in this analysis were unlikely to affect the likelihood of publication – there can be no negative/null result (Wang, 2018). Nevertheless, the Peter's test was used to test for bias for the purposes of this study, and the results of three Peter's tests (Hunter et al., 2014) are included as additional data (Appendix A).

## 3 Results

### 3.1 Comparison of markers of Lyme borreliosis risk in Scotland and Ireland

#### 3.1.1 Nymphal infection prevalence (NIP)

**Fig. 3:**
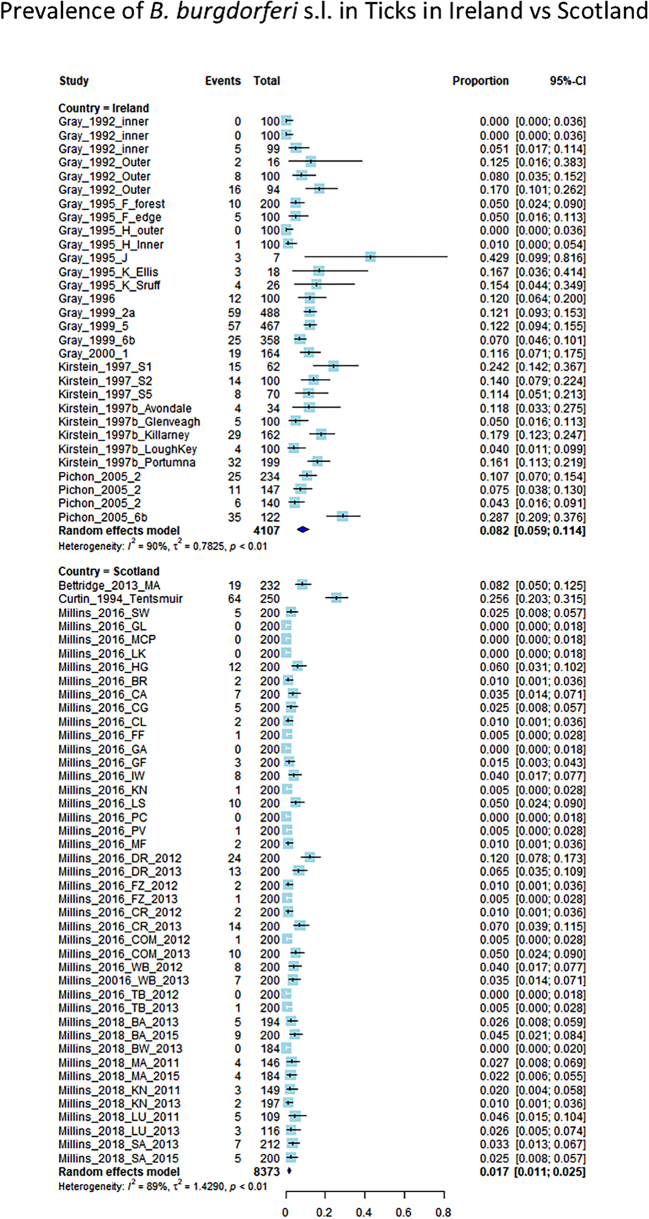
Forest plot comparing the NIP of *Borrelia burgdorferi* s.l. between Ireland and Scotland. Each study is represented as a box flanked by a horizontal line indicating the 95% confidence interval for the data. The overall pooled proportion is represented as a diamond. Proportions are displayed numerically as a proportion of 1. The overall NIP for Irish study sites is 8.2% (95% CI 5.9–11.4%) and is 1.7% (95% CI 1.1–2.5%) for Scottish sites.

The overall nymphal infection prevalence (*B. burgdorferi* s.l. complex) was higher in woodland sites in Ireland (8.2% (95%CI 5.9–11.4%), heterogeneity = 90%, *n* = 4107, 30 sites from 8 papers) than in Scotland (1.7% (95%CI 1.1–2.5%), heterogeneity = 89%, *n* = 8373, 42 sites from 4 papers) (Fig. 3).

**Fig. 4:**
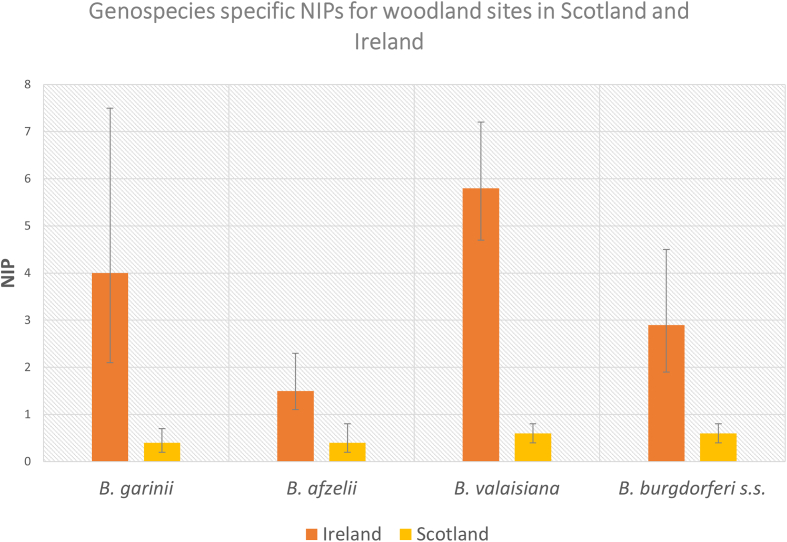
Comparison of NIP for all reported genospecies of *Borrelia burgdorferi* s.l. between Scotland and Ireland.

Four strains of *B. burgdorferi* s.l. were reported in both Ireland and Scotland: *B. garinii*, *B. afzelii*, *B. burgdorferi* s.s*.*, and *Borrelia valaisiana*. The overall prevalence of each individual strain was consistently higher in Ireland than in Scotland (Table 3, Fig. 4). Table 3 summarises the results of each meta-analysis of proportions with subgroup analysis (Forest plots for each meta-analysis in this section can be found in Appendix A). Strain-specific NIP data for Ireland were derived from 13 sites described in 5 papers, and strain-specific NIP data for Scotland were derived from 42 sites described in 3 papers.

**Table 3:** Summary of the outcomes of meta-analysis of proportions with subgroup analysis comparing the NIP of woodland sites in Ireland vs Scotland.

Strain	Ireland Prevalence meta-analysis outcome	Scotland Prevalence meta-analysis outcome	Ireland unweighted total infected ticks	Scotland unweighted total infected ticks
*Borrelia garinii*	4% (95% CI 2.1–7.5%), I^2^ = 87%, n = 1634	0.4% (95% CI 0.2–0.7%), I^2^ = 72%, n = 8125	98	67
*B. afzelii*	1.5% (95% CI 1.1–2.3%), I^2^ = 0%, n = 1634	0.4% (95% CI 0.2–0.8%), I^2^ = 87%, n = 8125	25	93
*Borrelia valaisiana*⁎	5.8% (95% CI 4.7–7.2%), I^2^ = 6%, *n* = 1634	0.6% (95% CI 0.4–0.8%), I^2^ = 7%, *n* = 8125	85	21
*Borrelia burgdorferi* s.s.⁎	2.9% (95% CI 1.9–4.5%), I^2^ = 35%, n = 1634	0.6% (95% CI 0.4–0.8%), I^2^ = 17%, n = 8125	38	22

⁎ Inverse method used for meta-analysis. GLMM method used in all other cases.

#### 3.1.2 Density of infected nymphs (DIN)

A meta-analysis of proportions with subgroup analysis was performed to compare the density of infected nymphs (DIN), between Ireland and Scotland. The numerator for each site included in the meta-analysis was ([Density per m2] x [Infected ticks]) and the denominator was [n] – the number of ticks collected at each site.

**Fig. 5:**
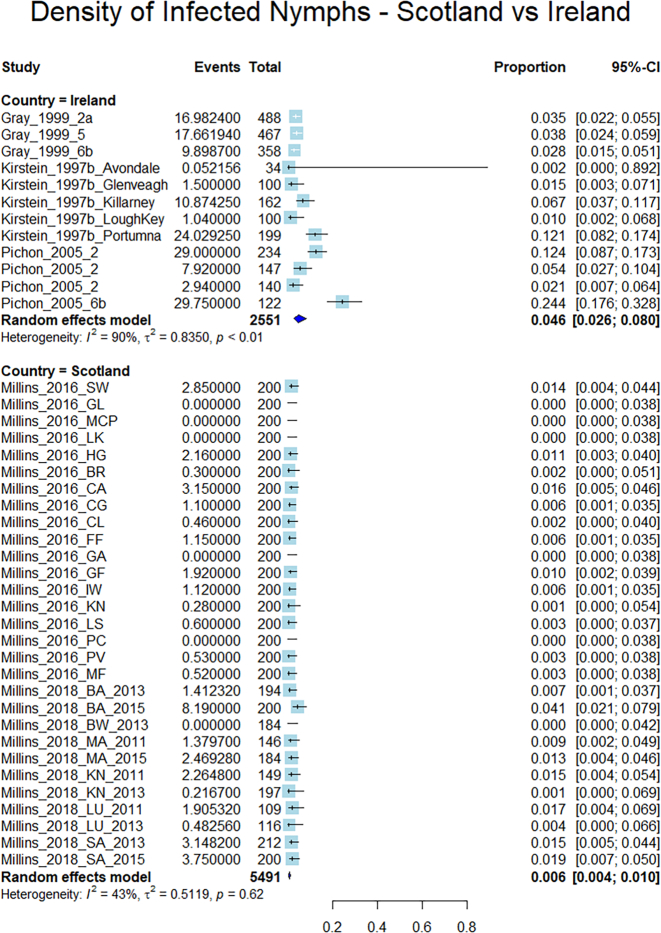
Forest plot showing a comparison of DIN between Scotland and Ireland. Each study is represented as a box flanked by a horizontal line indicating the 95% confidence interval for the data. The overall pooled proportion is represented as a diamond. Proportions are displayed numerically as a proportion of 1. The overall DIN for Irish study sites is 4.6%/m^2^ (95% CI 2.6–8%/m^2^) and is 0.6%/m^2^ (95% CI 0.4–1%/m^2^).

As was the case with NIP, the DIN for the Ireland subgroup (4.6% per m^2^, 95% CI 2.6–8%, heterogeneity = 90%, data from 12 sites from 3 papers, *n* = 2551) was significantly higher than that of the Scottish subgroup (0.6% per m2, 95% CI 0.4–1%, heterogeneity = 43%, data from 29 sites from 2 papers, *n* = 5491) (Fig. 5).

Note that the density was converted from Density/10m^2^ to Density/m^2^ before performing the meta-analysis. The reason for this was that the meta-analysis could only be performed when the numerator was a smaller number than the denominator. For consistency, all density-related results have been reported in m^2^ (rather than 10m^2^).

Taking into account varying levels of heterogeneity, this set of meta-analyses indicates a consistently higher NIP across four strains of *B. burgdorferi* s.l*.*, a higher overall NIP for *B. burgdorferi* s.l. complex as a group, and a higher DIN in Ireland than in Scotland.

### 3.2 The effect of habitat factors

Data on woodland site type (coniferous, mixed, deciduous), woodland size, and deer density were incorporated into separate meta-analyses on *B. burgdorferi* s.l. complex infection prevalence in nymphal ticks. In some, but not all cases, these factors could also be assessed against DIN and against individual strains of *B. burgdorferi* s.l. (Appendix A).

#### 3.2.1 Woodland type

**Fig. 6:**
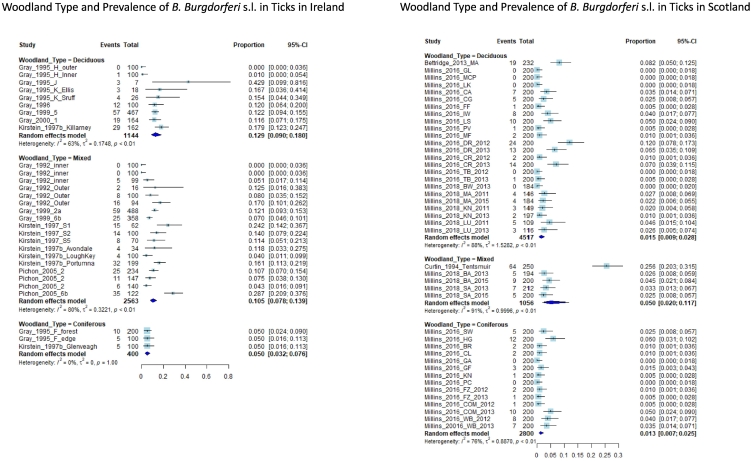
Forest plots showing the effect of woodland type on *Borrelia burgdorferi* s.l. NIP. Each study is represented as a box flanked by a horizontal line indicating the 95% confidence interval for the data. The overall pooled proportion is represented as a diamond. Proportions are displayed numerically as a proportion of 1. The forest plot on the left shows the difference in NIP between deciduous (12.9% (95% CI 9–18%)), mixed (10.5% (95% CI 7.8–13.9%)), and coniferous (5% (95% CI 3.2–7.6%)) sites in Ireland. The forest plot on the right shows the difference in NIP between deciduous (1.5% (95% CI 0.9–2.8%)), mixed (5% (95% CI 2–11.7%)), and coniferous (1.3% (95% CI 0.7–2.5%)) sites in Scotland.

The overall NIP was significantly lower in Irish coniferous woodlands (5%, 95% CI 3.2–7.6%), compared with mixed (10.5%, 95% CI 7.8–13.9%) and deciduous (12.9%, 95% CI 9–18%) woodlands (Fig. 6).

The DIN did not vary between woodland types in Ireland, however, it should be stressed that the dataset for this subgroup meta-analysis came from only three papers (Pichon et al., 2005; Gray et al., 1999; Kirstein et al., 1997b), the deciduous and coniferous subgroups only contained 2 and 1 datapoints respectively, and the mixed group contained studies with heterogenous outcomes (Appendix A). Based on this, it was decided that this subgroup meta-analysis was less robust than was desirable due to lack of input data, and the authors relied instead on results pertaining to NIP. Similarly, there was not enough data to report on genospecies-specific outcomes for coniferous sites, but there were no statistically significant differences between deciduous and mixed sites for NIPs of any of the genospecies studied (Appendix A).

The NIP for *B. burgdorferi* s.l. complex was also lowest in coniferous sites in Scotland (1.3% (95% CI 0.7–2.5%), I^2^ = 76%, 14 sites from 1 paper). The NIP was lower in coniferous sites than in mixed (5% (95% CI 2–11.7%, I^2^ = 91%, 5 sites from 2 papers) and deciduous sites (1.5% (95% CI 0.9–2.8%, I^2^ = 88%, 24 sites from 3 papers), but not significantly so (Fig. 6). For Scotland, there were only enough data to compare woodland type to overall NIP – meta-analyses comparing woodland type to DIN and to strain-specific NIP were not performed and are therefore not included in the appendix (see Table 2).

#### 3.2.2 Woodland size

**Fig. 7:**
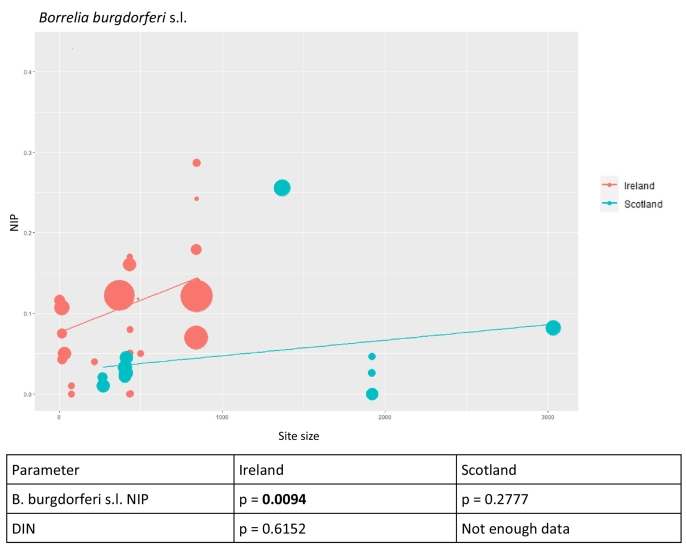
Bubble plot representing the effect of woodland size on NIP in sites in Ireland vs Scotland. Each study is represented by a ‘bubble’, the size of which is proportional to the study power. A regression line shows the relationship between the size of the woodland and NIP. The table beneath the bubble plot shows the *p*-values for meta regressions demonstrating the relationship between site size and NIP in Ireland (*p* = 0.0094) and Scotland (*p* = 0.2777), and between site size and DIN in Ireland (*p* = 0.6162) and Scotland (not enough data).

Scottish woodlands included in the dataset were on average larger than Irish woodlands (Fig. 7). Separate meta-regressions were performed comparing site size to NIP for Scotland and Ireland. A meta-regression comparing site size to DIN was also performed, but on the Irish site data (Fig. 7) only, for the reasons cited in the methodology.

The overall *B. burgdorferi* s.l. complex NIP increased with increasing site in Irish and Scottish woodlands, but was only significant for Irish woodlands (Fig. 7). When each genospecies was looked at individually, NIP tended to increase with increasing site size for all sites in Ireland, for all strains except for *B. afzelii*, though this increase was only significant for *B. burgdorferi* s.s*.* (Appendix A). None of the genospecies reported at Scottish sites displayed a significant relationship between NIP and site size, though in most cases there was again a trend towards increasing NIP with increased site size. The exception was *B. burgdorferi* s.s, which, in contrast to the Irish sites, showed a non-significant trend towards decreasing NIP with increased site size in the Scottish sites studied. (Appendix A).

#### 3.2.3 Deer density

**Fig. 8:**
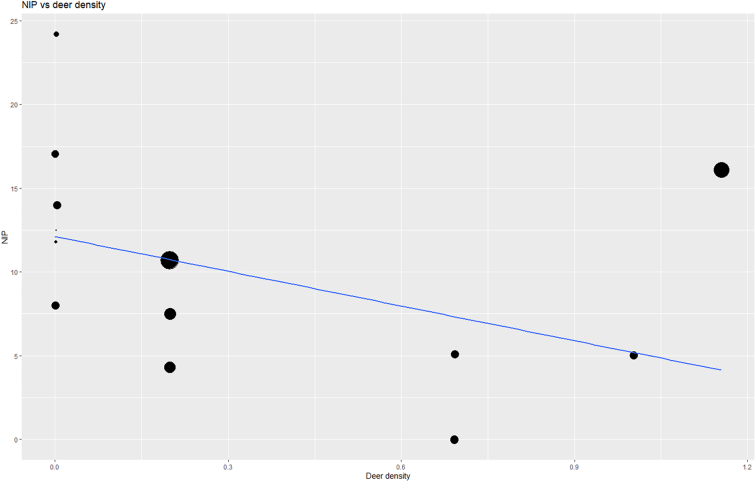
Bubble plot representing the relationship between NIP of *Borrelia burgdorferi* s.l. and deer density in study sites in Ireland. Each study is represented by a ‘bubble’, the size of which is proportional to the study size. A regression line shows the relationship between deer density and NIP.

Deer were the only hosts reported on frequently enough in the selected papers that their impact on infection in ticks could be included in the meta-analysis. However, information on deer density/abundance was collected or presented in different ways by different authors, particularly in the studies covering the Irish sites, and thus this data may be less reliable in nature than that of the other meta-analyses. Where deer data was included in studies of Scottish sites, these data were already incorporated into answering a similar question to that of this meta-analysis. Therefore, running a meta-analysis on these data would not add new information. Furthermore, only two studies on Scottish sites provided site-specific data on deer. Therefore, a meta-regression comparing deer density to NIP was run for Irish sites only. These data pertained to three deer species: fallow (*Dama dama),* red (*Cervus elaphus),* and sika deer (*Cervus nippon*)*.* There was a trend towards decreasing NIP with increasing deer density, which was non-significant (*p* = 0.113, Fig. 8). This remained the case when all genospecies were looked at individually (Appendix A).

The two Scottish studies which were eligible for inclusion and which reported on deer density (Millins et al., 2016; Millins et al., 2018) did not find a relationship between deer density and NIP (Millins et al., 2016; Millins et al., 2018) or tick abundance (Millins et al., 2018).

## 4 Discussion

The hard tick *I. ricinus* is an important vector of zoonotic pathogens in Europe (Zintl et al., 2017). This paper focuses on the *B. burgdorferi* s.l. complex – the pathogen species complex which causes Lyme borreliosis, the most common zoonotic tick-borne disease in Europe (Vandekerckhove et al., 2021; Lindgren and Jaenson, 2006). The reporting of neuroborreliosis at European level, and the epidemiological data on Lyme borreliosis cases collected in some countries allow for the surveillance of this infectious disease. However, the ability to supplement epidemiological data with environmental parameters of disease risk, such as NIP and DIN (LoGiudice et al., 2008; LoGiudice et al., 2003; Ostfeld and Keesing, 2000; Mysterud et al., 2016; Feldman et al., 2015), would improve surveillance capacity and add additional information on disease risk (van den Wijngaard et al., 2017). This study therefore used NIP, and – to a lesser extent - DIN as surrogate markers of disease-risk (LoGiudice et al., 2008; LoGiudice et al., 2003; Connally et al., 2006) to compare two similar North-Western European landscapes (Scotland, Ireland), and to quantify the impact of environmental factors including habitat type, size, and the presence of host species on disease risk. This study also appraised the usefulness of meta-analytic techniques in the field of disease ecology with a view to promoting standardised reporting methods for future meta-analytic studies thereby enhancing our knowledge of the disease.

The primary aim of this study was to undertake a structured literature review and meta-analysis to compare environmental markers of disease risk (NIP, DIN) between Scotland and Ireland. We found a weighted overall prevalence of *B. burgdorferi* s.l*.* in nymphal ticks in woodland sites in Ireland, based on a large population of ticks (*n* = 4107), of 8.2% (95% CI: 5.9–11.4%). Interestingly, this infection prevalence was higher than the weighted overall prevalence in Scottish sites, with an NIP of just 1.7% (95% CI 1.1–2.5%, *n* = 8373).

The same four strains of *B. burgdorferi* s.l. complex were reported for both countries – *B. garinii, B. afzelii, B. valaisiana,* and *B. burgdorferi* s.s*.*, with *B. valaisiana*, which is not a significant human pathogen (Zintl et al., 2017), accounting for a larger proportion of infected ticks in Ireland than in Scotland. When the NIP of each individual strain was measured via separate meta-analyses, the NIP for each was consistently significantly higher in Ireland than in Scotland. Though the authors acknowledge that density data for ticks are notoriously difficult to quantify using blanket-dragging methods, and thus density data should be interpreted with caution, available data showed that the density of ticks did not differ significantly between countries (*p*-value, 0.2125), and accordingly, the DIN for included sites was significantly higher in Ireland (4.6% per m^2^, 95% CI 2.6–8%, heterogeneity = 90%, *n* = 2551, data from 12 sites from 3 papers) than in Scotland (0.6% per m2, 95% CI 0.4–1%, heterogeneity = 43%, *n* = 5491, data from 29 sites from 2 papers). All analyses performed in this study indicate that the examined markers of disease risk were higher in Ireland than in Scotland. This is interesting because, as previously mentioned, the estimated incidence of Lyme borreliosis based on human epidemiological data is considerably higher in Scotland than in Ireland. It is important to stress that this study relies exclusively on environmental data, and does not take human factors (e.g. risk awareness, levels of outdoor space use) into account. However, even bearing this caveat in mind, the results imply that further investigation is needed on the incidence of Lyme borreliosis in Scotland and Ireland, and highlights the utility of incorporating environmental markers into disease risk surveillance.

Of note, the papers which provided data for sites in Ireland (Pichon et al., 2005; Gray et al., 2000; Gray et al., 1999; Kirstein et al., 1997a; Kirstein et al., 1997b; Gray et al., 1995; Gray et al., 1992; Gray et al., 1996) tended to be older (1992–2005) than those which provided data for sites in Scotland (Millins et al., 2016; Curtin and Pennington, 1994; Millins et al., 2018; Bettridge et al., 2013) (1994–2018). As well as highlighting a gap in the more recent literature on environmental factors influencing NIP in Ireland, it could also be inferred that the date of sample collection had a bearing on the results of this analysis. However, a recent paper (Zintl et al., 2020) concluded that the distribution and *B. burgdorferi* s.l. infection prevalence of nymphal ticks in Ireland has not changed since the 1990s. This provides some reassurance that data that was incorporated into this analysis from older papers remains reliable.

The authors note that the NIP for Scotland as calculated by this meta-analysis (1.7% (95% CI 1.1–2.5%), I^2^ = 89%) was lower than a previous study by James et al. *(*James et al., *2013**)*, which put the average NIP of 25 Scottish sites at 5.6% (±1%, range 0.8–13.9%). These differences in outcomes may be accounted for by geographic site differences, in that James et al. concentrated on more northerly areas of Scotland, whereas the meta-analysis in this study also included several sites from the southern half of the country, potentially reflecting a difference in NIP in different areas of Scotland. Similarly, a recent study of Irish sites (Zintl et al., 2020) put the overall NIP for woodland sites in Ireland at 14.2% (CI: 11.4–17.0%) compared to 8.2% (95% CI: 5.9–11.4%) in this study, though the study authors noted that a particularly high NIP in one location (Muckross Demesne, Killarney) brought this average up. The site in question was also included in the dataset of the current study, but the weighting of sample sizes inherent to the meta-analysis method reduces the influence that individual datasets from certain sites have on the overall pooled effect size, which is one of the many benefits of meta-analysis that is particularly applicable to the setting of disease ecology.

The second aim of this meta-analytic study was to assess the impact of habitat factors described in the literature on disease risk markers in Ireland and Scotland. These countries were chosen as examples of host-species poor regions in North-Western Europe. The selected articles provided sufficient data to assess the following parameters: woodland type, woodland size, and deer density. As mentioned, the power of a meta-analysis is highly dependent on the type of data reported in the literature. Many sub-studies were excluded and some analyses could not be run due to lack of reporting of site-specific data, usually because a site-specific analysis was not relevant in the context of the original study.

The subgroup-analysis which looked at woodland type as a factor affecting infection prevalence found that the NIP was lowest in coniferous sites. This was true for sites in both Scotland and Ireland – though there were overlaps between confidence intervals, indicating that these differences were not statistically significant. The fact that overall NIP was lowest in coniferous sites is in keeping with a Scottish study, which found that NIP was higher in mixed/deciduous than coniferous woodlands (James et al., 2013). Furthermore, a Swedish study reporting on the Normalised Difference Vegetation Index (NDVI), which measures the photosynthetic ability of a woodland, and which is higher in deciduous than in coniferous sites, found that higher NDVI was correlated with higher tick abundance and higher human disease risk (Pfäffle et al., 2013). There were not enough data to provide DIN or strain-specific NIP outcomes on how coniferous sites compared with mixed and deciduous sites. This was due, in part, to a lack of reporting on tick density data or genospecies specific data for each individual site in a given paper.

Meta-regressions were performed to assess the relationship between woodland size and NIP. It was noted that the size of woodland sites included for Scotland were on average much greater than those in Ireland. Ireland is a more fragmented landscape than Scotland, meaning that woodlands in Ireland tend to be smaller and more separated from each other (EEA, 2022). Nevertheless, there was a significant increase in overall (*B. burgdorferi* s.l. complex) NIP with increasing site size in Irish woodlands and a trend towards increasing NIP with increasing site size in Scottish woodlands. This differs from US data, which indicate that rates of infected nymphs and human Lyme borreliosis rates are both inversely related to habitat fragment size (LoGiudice et al., 2008; Allan et al., 2003). When individual strains were examined in this analysis*,* all except one showed trends towards increasing NIP with increasing woodland size. The exception was *B. burgdorferi* s.s. which is the dominant strain in North America (Kilpatrick et al., 2017), but which accounts for only a fraction of the positive ticks in this study's dataset. *B. burgdorferi* s.s*.* NIP increased significantly with increasing woodland size in sites in Ireland but showed a non-significant inverse relationship with woodland size in Scottish sites. There is evidence to suggest that habitat fragmentation has greater impact when assessed at regional landscape level, rather than in the context of the size of an individual woodland/habitat (Kilpatrick et al., 2017; Pfäffle et al., 2013), which may explain both why the overall NIP is higher in Ireland (a country with a more fragmented landscape) than in Scotland (which has a less fragmented landscape) and why there was no inverse relationship between overall NIP and habitat size for the sites we analysed. Therefore, it is possible that the relationship between habitat size and NIP is non-linear at the extremes of site size, and to address this question, more studies are needed which incorporate highly fragmented areas with very small woodland sites, and areas with larger continuous woodland.

The dilution effect which theorises that as host species richness increases, NIP decreases (LoGiudice et al., 2008; Ostfeld and Keesing, 2000; Mysterud et al., 2016) was developed and tested in the USA. However, the relationship between host species richness and NIP/human Lyme borreliosis risk in the European disease system needs to be explored further (Mysterud et al., 2016). Unfortunately, data on host species richness could not be incorporated into this meta-analysis due to the lack of a standardised approach to the assessment of vertebrate hosts across studies. Differences in host species availability may be a key factor in the abovementioned patterns of the strain-specific NIPs.

Deer density were the only host data included in this meta-analysis. Deer are considered a reproduction host for ticks, with the presence of deer expected to increase tick abundance (Gilbert, 2016; Millins et al., 2017). However, deer are also thought to be incompetent hosts for *B. burgdorferi* s.l. (Kilpatrick et al., 2017; Gilbert, 2016), meaning areas with a high deer abundance may have a lower NIP. Indeed, European studies have reported differing relationships between deer and NIP, with some reporting a negative relationship, while others report a positive or no relationship (Gilbert, 2016). This study observed a non-significant trend towards decreasing NIP with increasing deer densities in Irish sites, which was true for all analysed genospecies. This finding was in agreement with those of some of the studies included in the meta-analysis itself (Gray et al., 1992; Gray et al., 1999; Kirstein et al., 1997a). A meta-analysis was not run on the relationship between deer density and NIP in Scottish sites: two of the studies which met the inclusion criteria for analysis (Millins et al., 2016; Millins et al., 2018), were multi-site studies with a wide geographical spread which had addressed this question, and which had found no relationship between deer density and NIP. As mentioned, several European studies have cited complex relationships between deer density and tick densities/NIP (Gilbert, 2016). Therefore, a larger dataset on deer densities, tick densities, and NIP in Europe is needed to fully answer the question of the relationship between deer density and Lyme borreliosis risk. Datasets incorporating both deer and tick abundances as well as NIP in future meta-analyses would likely shed more light on the relationship. Furthermore, more landscape-scale studies are needed to identify how host diversity and other host factors affect NIP in the European disease system. As yet, the question of how host factors influence markers of Lyme borreliosis risk in a species-poor region of North-Western Europe remains unanswered.

The third aim of this study was to appraise the use of proportion meta-analysis with subgroup analysis as a tool for use in the field of disease ecology.

Meta-analysis is considered a gold standard technique for evidence synthesis in medicine (Haidich, 2010). The specific technique of meta-analysis of proportions with subgroup analysis is most often applied in medicine and epidemiology (Wang, 2018), as it lends itself to the examination of disease rates in a population. In the realm of disease ecology, often the rate of infection in a disease vector is of central importance, so it stands to reason that meta-analysis of proportions is a statistical technique that should be applied within this field for evidence synthesis. Though other statistical methods have been used heretofore to combine multiple studies in this area (Strnad et al., 2017; Estrada-Peña et al., 2018; Estrada-Peña et al., 2011), this is the first time, to the authors' knowledge, that a study-weighted meta-analysis of proportions with subgroup-analysis has been used to assess the impact of environmental factors on infection prevalence in ticks. While the present study used the method to explore infection rates with *B. burgdorferi,* it is also applicable to other tick-borne pathogens.

However, despite its many strengths as a statistical tool, there are also important limitations. The main drawback of the use of meta-analysis of proportions in disease ecology is the fact that it is dependent upon high levels of detail and consistency in data-reporting within the published literature. For example, if site-specific data pertaining to infection prevalence and number of vector samples collected (site-specific n) are not provided by the authors, a paper should be excluded, whereas it may have been included in a traditional meta-analysis of proportions used in epidemiology, where site-specific influences and characteristics are less important variables.

Furthermore, data on density are often important in interpreting risk in disease ecology (for example, DIN in this study), whereas density is not included in a traditional meta-analysis in epidemiology. The ‘metaprop’ function in R (Viechtbauer, 2010), while excellent and highly versatile, cannot compute an overall effect if the denominator is higher than the numerator – which can be the case for density measures such as DIN. In this study, the authors were able to adjust the unit of density (to m^2^ rather than 10m^2^) to overcome this limitation, but this may not be the case for all datasets.

Finally, meta-analyses of proportions use observational (i.e. non-interventional) rather than comparative statistical methods, to obtain an accurate prevalence measurement (Wang, 2018). While this is of great use in epidemiology and public health studies (Wang, 2018), it should be noted that a high (>75%) degree of heterogeneity is usually found in meta-analyses of proportions (Borenstein, 2009), and thus the outcomes must be interpreted with a degree of caution.

The strength of this statistical technique lies in its ability to combine measures of effect size from multiple studies onto one measurement scale, allowing for the synthesis of a robust effect measure with a higher n-value than might be achieved by an individual study, while taking individual study power into account, thus reducing the under- or overrepresentation of small/large studies (Koricheva et al., 2017). It is therefore a powerful tool for use in disease ecology, where study size is highly dependent on the number of sites involved and the sample collection capacity (i.e. the ability to collect a vector and test it for the presence of the pathogen). Where meta-analyses are applied more regularly to disease ecology, there is an opportunity to generate standardised data which supplements the information that can be gathered on disease risk in countries/regions. Using meta-analysis to gather information on disease vectors and on the environmental variables affecting these vectors adds valuable information on diseases of concern to public health and animal health. However, this relies on sample collection and reporting being conducted in a highly standardised manner.

### 4.1 Recommendations

While it is acknowledged that researchers working on disease vectors including *I. ricinus* may have different research questions, the authors have identified several gaps in the literature which, if addressed, would help in conducting future meta-analyses in disease ecology or which would answer questions pertaining to Lyme disease risk in North-Western Europe (Table 4).

**Table 4:** Gaps in the literature and recommendations for future research.

Gap identified	Recommendation	Example
Many papers were excluded from the current analysis due to lack of site-specific reporting of data (e.g. only aggregate data were reported in several papers).	All papers published pertaining to data on disease vectors / infection prevalence in disease vectors should make site-specific data available as standard or as an additional dataset.	Papers on ticks collected from several sites shall report data from each site including tick density, NIP, for each site, site type, coordinates.
Methods for collecting the disease vector occasionally varied between papers	Vectornet standards (Vectornet, 2018) for sample collected should be applied in all future studies of disease vectors.	Ticks shall be collected via blanket dragging with a 1m^2^ white material, over 30 × 5 m drags, with 5 m between each drag.
Reporting of data on important hosts for disease vectors is sparse, and sampling methods for same are heterogenous. According to Mysterud et al. (Mysterud et al., 2016), the effect of vertebrate host diversity on Lyme borreliosis risk has not been tested outside of North America.	A standard methodology could be developed specifically for the survey of wildlife hosts of vector borne diseases. If authors of European studies report more often on disease vector hosts, a meta-analytic approach can be used to answer the question of whether host diversity influences Lyme borreliosis risk for the first time.	Data on tick hosts shall be recorded via a global biodiversity information form.
There is a limitation in the ability to incorporate density data into meta-analyses of proportions	Development of an accessible way to incorporate disease vector density data into a prevalence meta-analysis package for direct application to disease ecology.	DIN can be calculated via meta-analysis package, even in cases where the denominator exceeds the numerator.

## 5 Conclusion

This study used meta-analyses of proportions to compare environmental markers of Lyme borreliosis risk between two countries in North-Western Europe. The aim was to compare disease risk in two neighbouring countries which are ecologically very similar but where the estimated incidence of Lyme borreliosis is difficult to compare using existing epidemiological data. The NIP and DIN in the woodland sites included in our study were both higher in sites from Ireland than Scotland, suggesting a need to re-examine and further explore estimated incidences in humans in both countries. This study also showed that meta-analysis can be a viable method with which to analyse data on the environmental indicators of disease risk, generating information which can supplement epidemiological data to assess disease risk in different countries.

This study also used meta-analyses of proportions to investigate the relationship between various environmental variables and disease risk. Of the three woodland site types examined, the lowest NIP was found in coniferous sites. Although the relationship between site size and NIP varied between strains, there was a significant increase in overall NIP with increasing site size in Ireland, but not in Scotland, which may be accounted for by the highly fragmented landscape of Ireland and small woodland stand sizes. While deer densities did not have a significant effect on overall NIP, there was a trend towards decreased NIP with increasing deer abundance in woodland sites in Ireland. These outcomes illustrate that meta-analysis of proportions is a useful tool with which to explain the environmental variables impacting disease risk.

Overall, the authors note that with a standardised approach to data collection, a meta-analysis of proportions with subgroup-analysis is an excellent method with which to investigate zoonotic disease prevalence in a disease vector. In particular, due to the ability to make robust comparisons between countries akin to that of a highly resourced international study, this approach can be in principle be applied to any tickborne pathogen, and has the potential to be used in a pan-European context thus bridging the gap between disease ecology and epidemiology. Using meta-analysis to gather information on disease vectors adds valuable information on diseases of public health concern, increasing our ability to survey and control vector-borne zoonoses. To achieve this, the authors would recommend that the adoption of a standardised approach to data collection (where possible) be considered by those working in the field so that robust meta-analysis can be achieved in the future to further our understanding of this disease.

## Declaration of Competing Interest

The authors declare no conflicts of interest.

## Appendix A Supplementary data

Additional information regarding analyses of publication bias, genospecies-specific data, and NIP/DIN by location in Ireland. Image 1

Dataset used in the analyses Image 2
