# Supplementary material for: Meta-analysis: A useful tool to assess infection prevalence and disease ecology of Borrelia burgdorferi sensu lato in nymphal ticks in North-Western Europe with recommendations for a standardised approach to future studies
Source: Parasite Epidemiol Control. 2022 May 31;18:e00254. doi: 10.1016/j.parepi.2022.e00254 (PMC9167692; doi:10.1016/j.parepi.2022.e00254)
Supplement: Supplementary file 1 — Additional information regarding analyses of publication bias, genospecies-specific data, and NIP/DIN by location in Ireland. [file mmc1.docx]

# Appendix A

## Publication bias

### Peter’s test – all included data at site level

Tools for predicting publication bias in comparative meta-analyses, such as the funnel plot and Egger’s test, are considered less pertinent in the setting of meta-analyses of proportions than in comparative meta-analyses [51, 52]. Instead, a Peter’s test [52] was applied, which uses size rather than standard error to test for bias in a meta-analysis of proportions. Specifically, the Peter’s test uses a regression for the inverse of the sample size of the study which is then weighted by the total number of events vs non events. Peter’s tests were applied at site level for all sites included in the meta-analysis.
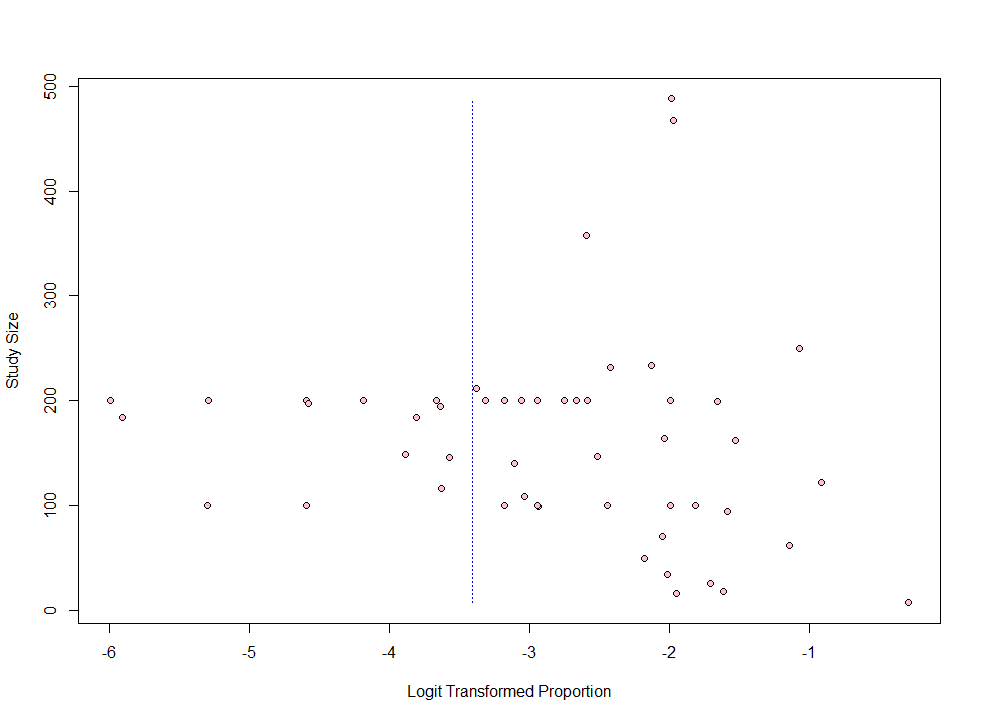


Figure 1 Funnel plot results showing study size vs log transformed proportions for all site-level data included in meta-analyses.

A linear regression test of funnel plot asymmetry (based on sample size) (Peter’s test) was applied to all site-level data included in the meta-analyses in this study. The resulting p-value was 0.1872, representing lack of significant asymmetry. Bias was therefore not detected.

### Peter’s test – included papers

For completeness, two further Peter’s tests were run. Figure 2a shows the results of a Peter’s test on a meta-analysis which incorporates the overall NIP reported by all papers included in this study. That is, the NIP at paper level for all sites in the paper, regardless of site type.


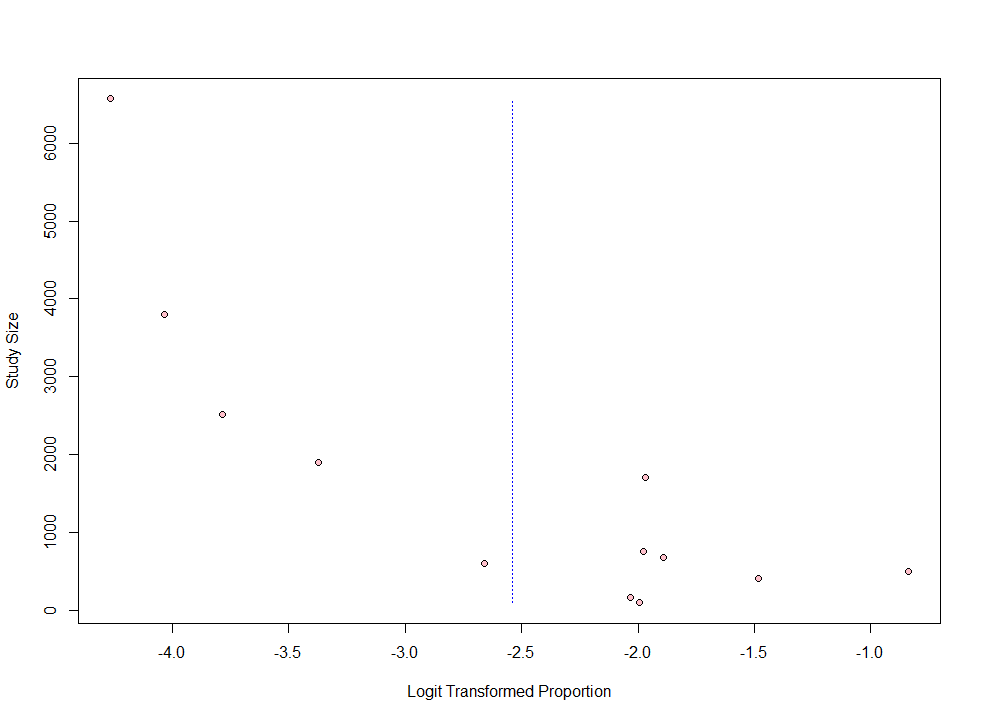


Figure 2a Funnel plot displaying study size vs log transformed proportions for all paper-level data included in meta-analyses

The resulting p-value was 0.1307, representing lack of significant asymmetry. Bias was not detected.

Figure 2b shows the results of a Peter’s test on a meta-analysis which incorporates the overall NIP at paper level for all woodland sites in this study. That is, the NIP for each woodland site in a paper was calculated and combined such that a woodland NIP was created for each paper at paper level. A meta-analysis was run on this.


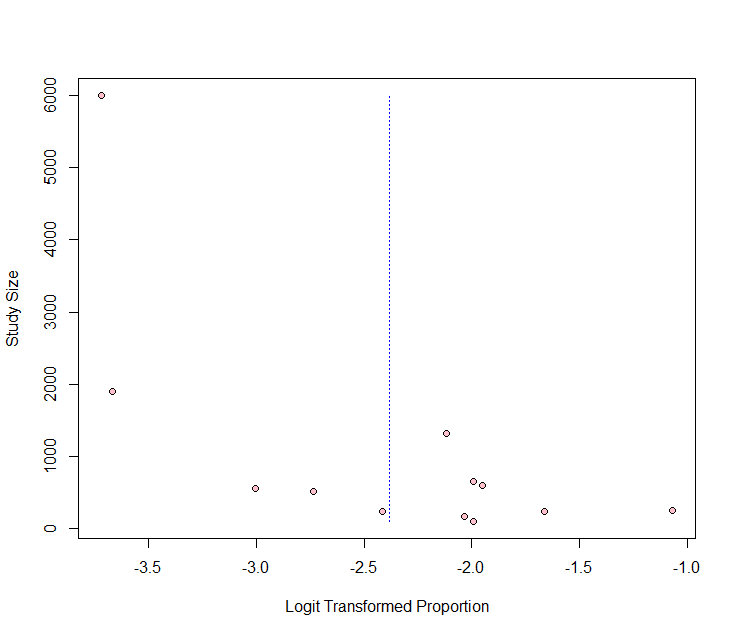


Figure 2b Funnel plot displaying study size vs log transformed proportions for all woodland NIP data at paper-level for papers included in meta-analyses

The resulting p-value was 0.04697, indicating significant asymmetry. The asymmetry here is thought to be due to the difference in prevalence between the sites from Scotland and Ireland, but the plot has been included for completeness.

## Genospecies-specific forest plots

The figures in this section are the individual forest plots representing the overall NIPs of each strain of *Borrelia burgdorferi* s.l. detected in Scotland and Ireland. These data are summarised in Table 3 and Figure 4 in the manuscript.

##
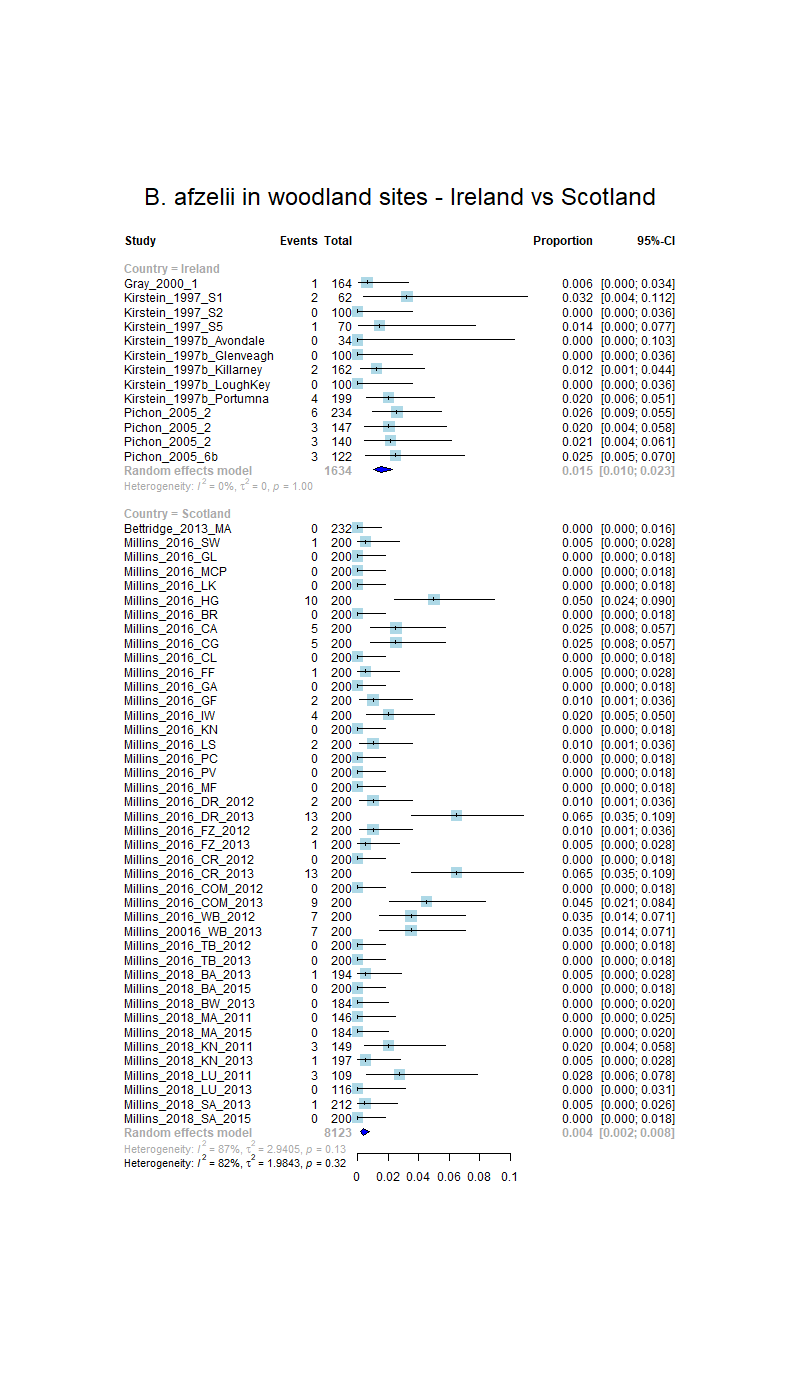


***B. afzelii* in woodland sites – Ireland vs Scotland**

Figure 3 Forest plot comparing B. afzelii prevalence in ticks in woodland sites in Ireland vs Scotland. This figure shows an overall weighted average prevalence of B. afzelii in woodland sites in Ireland of 1.5% (95% CI 1-2.3%), and of 0.4% (95% CI 0.2-0.8%) for sites in Scotland.

***B. garinii* in woodland sites – Ireland vs Scotland**


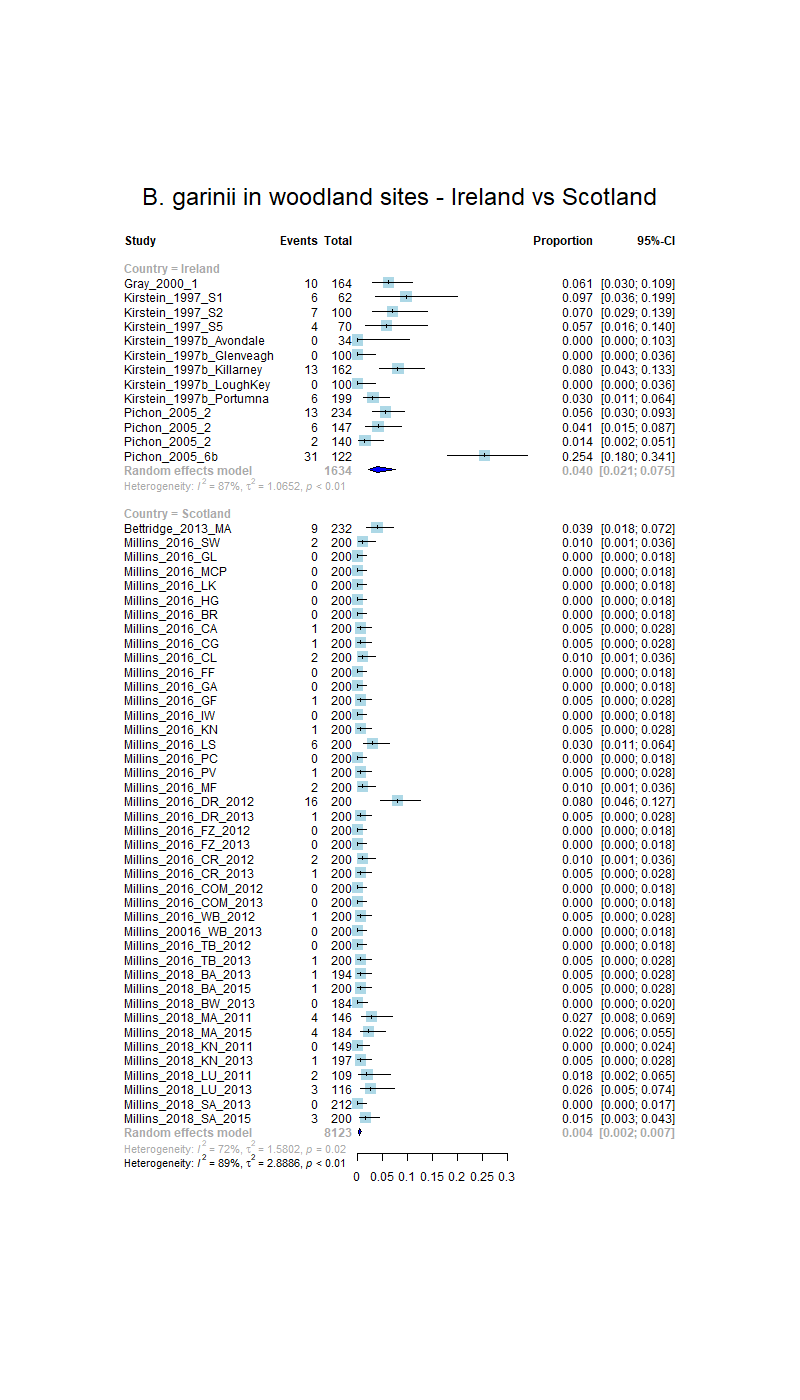


Figure 4 Forest plot comparing B. garinii prevalence in ticks in woodland sites in Ireland vs Scotland. This figure shows an overall weighted average prevalence of B. garinii in woodland sites in Ireland of 4% (95% CI 2.1-7.5%), and of 0.4% (95% CI 0.2-0.7%) for sites in Scotland.

***B. burdorferi* sensu stricto in woodland sites – Ireland vs Scotland**


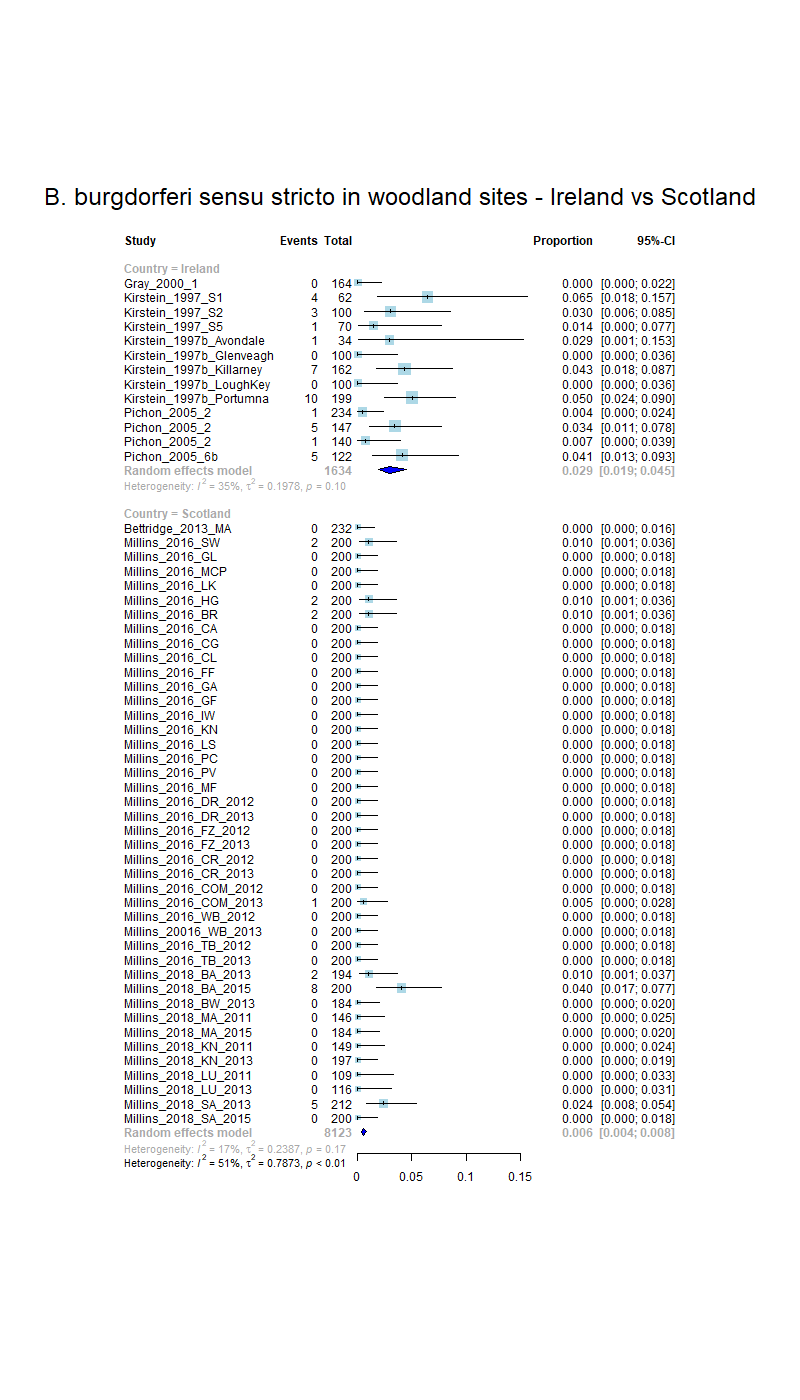


Figure 5 Forest plot comparing B. burgdorferi s.s. prevalence in ticks in woodland sites in Ireland vs Scotland. This figure shows an overall weighted average prevalence of B. burgdorferi s.s. in woodland sites in Ireland of 2.9% (95% CI 1.9-4.5%), and of 0.6% (95% CI 0.4-0.8%) for sites in Scotland.

***B. valaisiana* in woodland sites – Ireland vs Scotland**


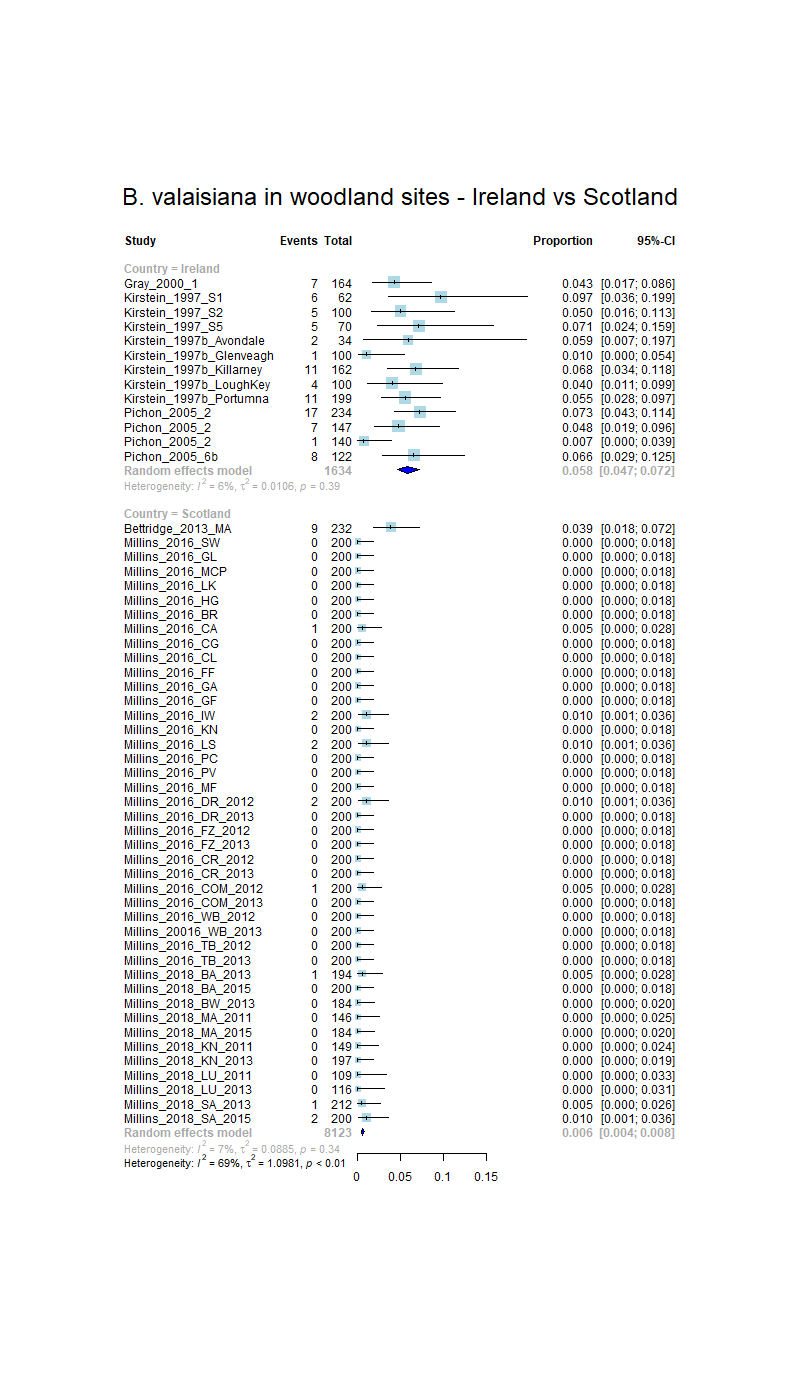


Figure 6 Forest plot comparing B. valaisiana prevalence in ticks in woodland sites in Ireland vs Scotland. This figure shows an overall weighted average prevalence of B. valaisiana in woodland sites in Ireland was 5.8% (95% CI 4.7-7.2%), and of 0.6% (95% CI 0.4-0.8%) for sites in Scotland.

## Genospecies-specific and DIN data for woodland-site type variables

The tables and figures in this section display the data that pertains to section 3.2.1 of the manuscript. Table 1 summarises the outcomes of meta-analyses with subgrouping by forest types for data from forests in Ireland. Meta-analyses were conducted individually on each strain found in Ireland, as well as for overall NIP and DIN. Figures 7-11 are forest plots for the outcomes of each individual analysis.

Table 1 Summary of outcomes of proportion meta-analyses with subgroup analysis assessing the effect of woodland type on NIP/DIN in woodland sites in Ireland

| Strain/other | Deciduous | Mixed | Coniferous |
| --- | --- | --- | --- |
| *B. garinii* | 7.1% (95% CI 4.7-10.4%), I^2^=0% | 4.2% (95% CI 2-8.4%), I^2^=86% | Not enough data |
| *B. afzelii* | 0.9% (95% CI 0.3-2.8%), I^2^=0% | 1.8% (95% CI 1.2-2.8%), I^2^=0% | Not enough data |
| *B. valaisiana* | 5.5% (95% CI 3.5-8.6%), I^2^=0% | 5.4% (95% CI 4.1-7%), I^2^=15% | Not enough data |
| *B. burgdorferi* s.s*.* | 0.7% (95% CI 0-24.1%), I^2^=77% | 2.2% (95% CI 1.2-4%), I^2^=54% | Not enough data |
| *B. burgdorferi* s.l. | 1.29% (95% CI 9-18%), I^2^=63% | 10.7% (95% CI 7.8-13.9%), I^2^=80% | 5% (95% CI 3.2-7.6%), I^2^=0% |
| DIN | 4.5% (95% CI 3.2-6.5%), I^2^=0% | 4.9% (95% CI 2.4-9.9%), I^2^=92% | Not enough data |

***B. garinii* NIP by woodland site type in Ireland**


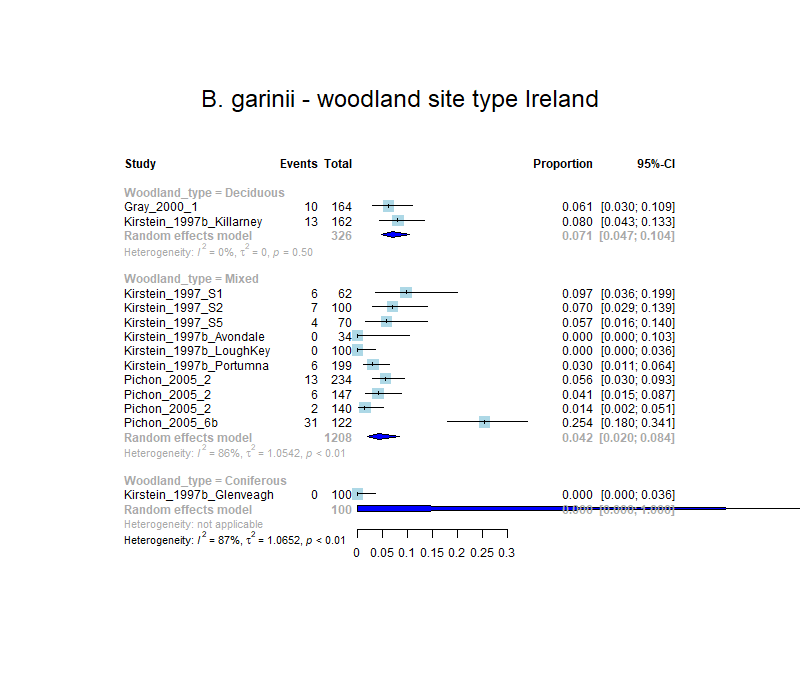


Figure 7 Forest plot showing B. garinii prevalence in nymphal ticks by woodland site type in Ireland. This figure shows an overall weighted average prevalence of B. garinii in deciduous sites in Ireland of 7.1% (95% CI 4.7-19.4%), of 4.2% (95% CI 2-8.4%) for mixed sites, and of 0% (95% CI 0-3.6%) for coniferous sites. However, as there was only one site in the coniferous subgroup, these data were excluded.

***B. afzelii* NIP by woodland site type in Ireland**


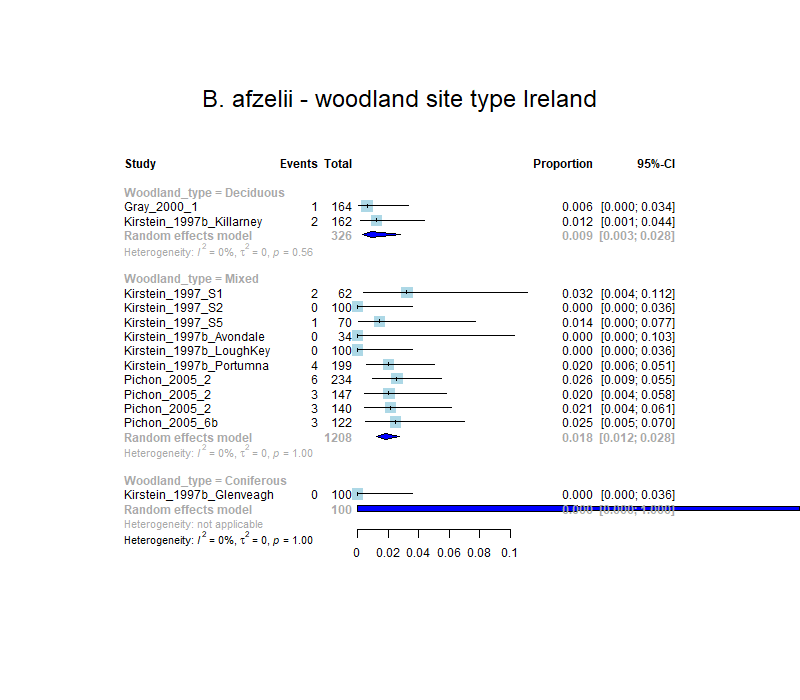


Figure 8 Forest plot showing B. afzelii prevalence in nymphal ticks by woodland site type in Ireland. This figure shows an overall weighted average prevalence of B. afzelii in deciduous sites in Ireland of 0.9% (95% CI 0.3-2.8%), of 1.8% (95% CI 1.2-2.8%) for mixed sites, and of 0% (95% CI 0-3.6%) for coniferous sites. However, as there was only one site in the coniferous subgroup, these data were excluded.

***B. valaisiana* NIP by woodland site type in Ireland**


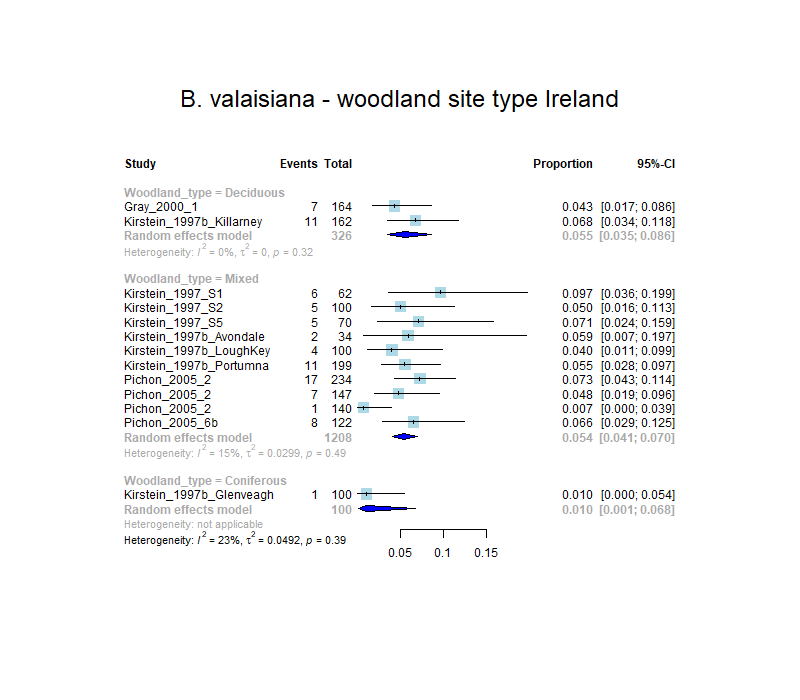


Figure 9 Forest plot showing B. valaisiana prevalence in nymphal ticks by woodland site type in Ireland. This figure shows an overall weighted average prevalence of B. valaisiana in deciduous sites in Ireland of 5.5% (95% CI 3.5-8.6%), of 5.4% (95% CI 4.1-7%) for mixed sites, and of 0% (95% CI 0.1-6.8%) for coniferous sites. However, as there was only one site in the coniferous subgroup, these data were excluded.

***B. burgdorferi* sensu stricto NIP by woodland site type in Ireland**


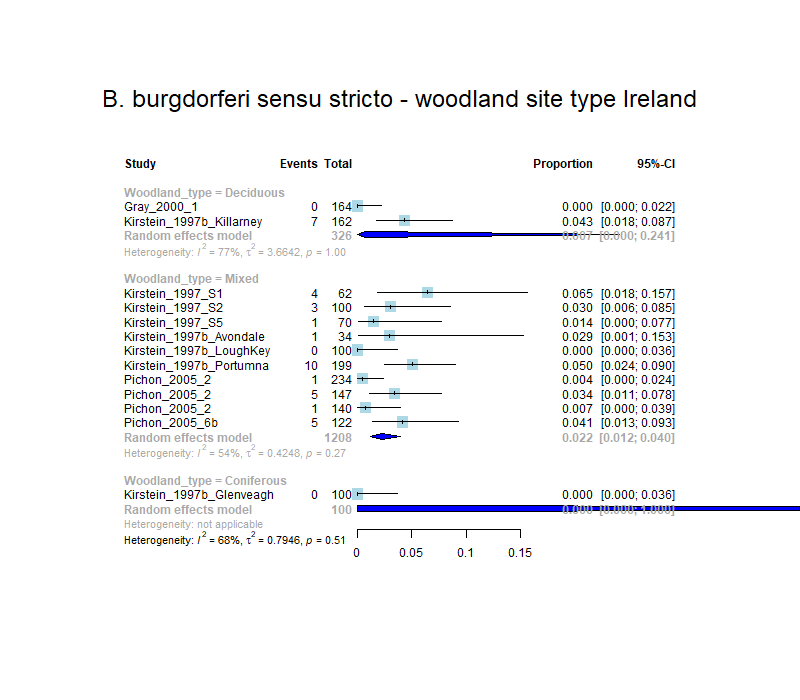


Figure 10 Forest plot showing B. burgdorferi s.s. prevalence in nymphal ticks by woodland site type in Ireland. This figure shows an overall weighted average prevalence of B. garinii in deciduous sites in Ireland of 0.7% (95% CI 0-24.1%), of 2.2% (95% CI 1.2-4%) for mixed sites, and of 0% (95% CI 0-3.6%) for coniferous sites. However, as there was only one site in the coniferous subgroup, these data were excluded.

**Density of infected nymphs by woodland site type in Ireland**


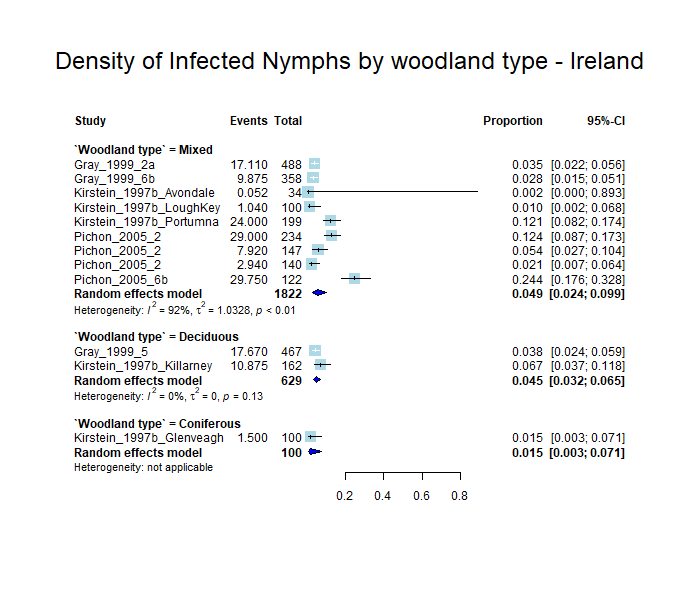


Figure 11 Forest plot showing DIN by woodland site type in Ireland. This figure shows an overall weighted average DIN in deciduous sites in Ireland of 4.9% (95% CI 2.4-9.9%), of 4.5% (95% CI 3.2-6.5%) for mixed sites, and of 1.5% (95% CI 0.3-7.1%) for coniferous sites. However, as there was only one site in the coniferous subgroup, these data were excluded.

## Genospecies-specific and DIN data for woodland-site size variables

The tables and figures in this section display the data that pertains to section 3.2.2 of the manuscript. Meta-regressions were conducted individually on each strain found in Ireland and Scotland, as well as for overall NIP and DIN. Figure 12 shows the bubble plots and p-values of meta-regressions comparing NIP/DIN to site size for data from forests in Ireland vs Scotland.

| 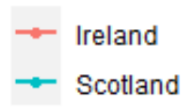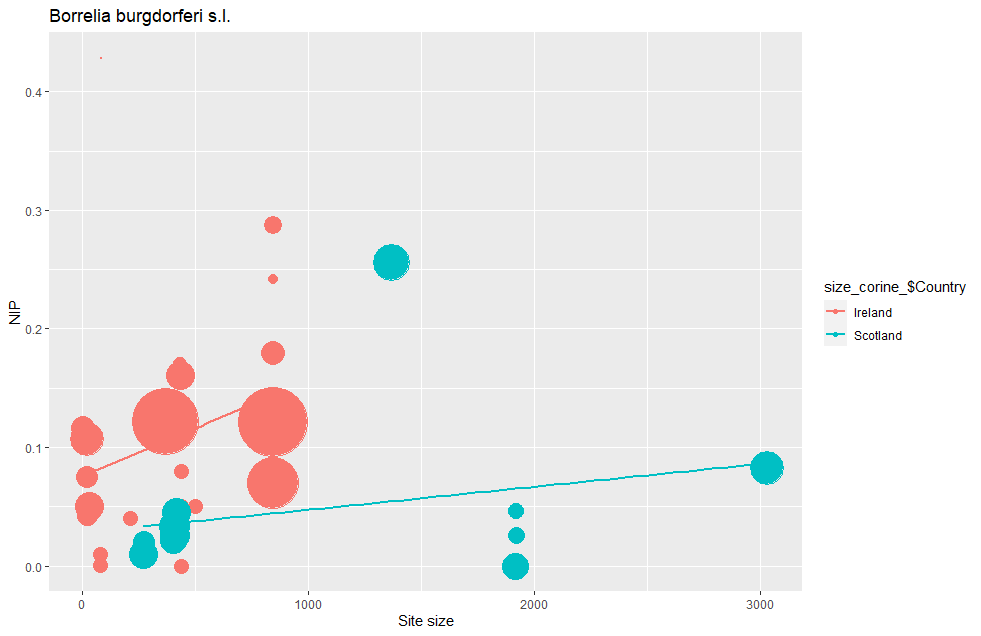 | |
| --- | --- |
| 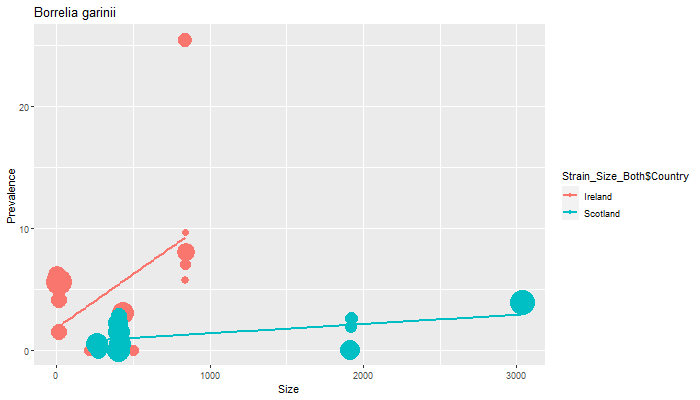 | 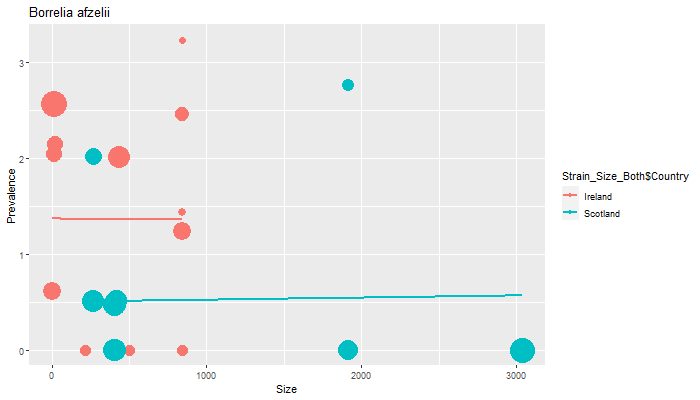 |
| 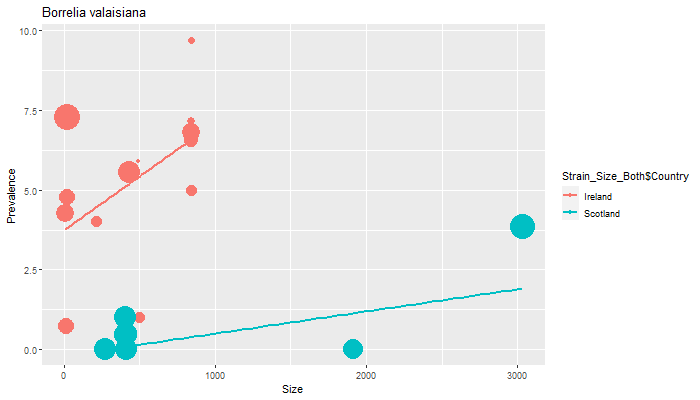 | 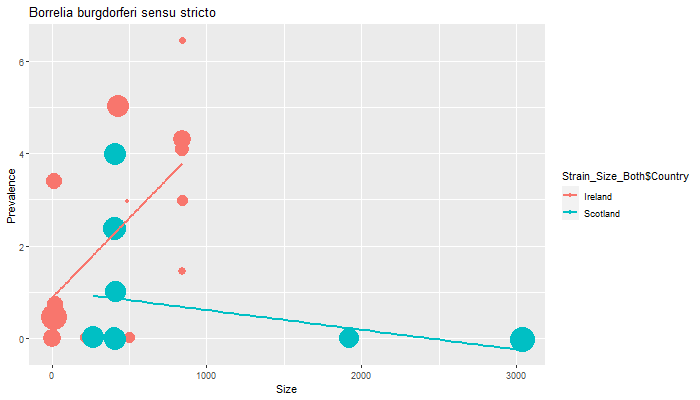 |
| \| Strain \| Ireland \| Scotland \| \| --- \| --- \| --- \| \| *B. garinii* \| p = 0.0585 \| p = 0.5782 \| \| *B. afzelii* \| p = 0.6614 \| p = 0.6974 \| \| *B. valaisiana* \| p = 0.1510 \| p = 0.4433 \| \| *B. burgdorferi* s.s. \| **p = 0.0156** \| p = 0.3677 \| \| Overall (*B. burgdorferi* s.l.) \| **p = 0.0094** \| p = 0.2777 \| \| DIN \| p = 0.6152 \| Not enough data \| | |

Figure 12 Bubble plots and p-values for DIN and for genospecies-specific NIPs by woodland site size in Ireland vs Scotland. A significant p-value demonstrates a significant relationship between site size and NIP.

## Genospecies-specific data for deer density and NIP – sites in Ireland only

The tables and figures in this section display the data that pertains to section 3.2.3 of the manuscript. Meta-regressions were conducted individually on each strain found in Ireland, as well as for overall NIP. Figure 13 shows the bubble plots and p-values of meta-regressions comparing individual strain NIPs to deer density data from forests in Ireland.

| 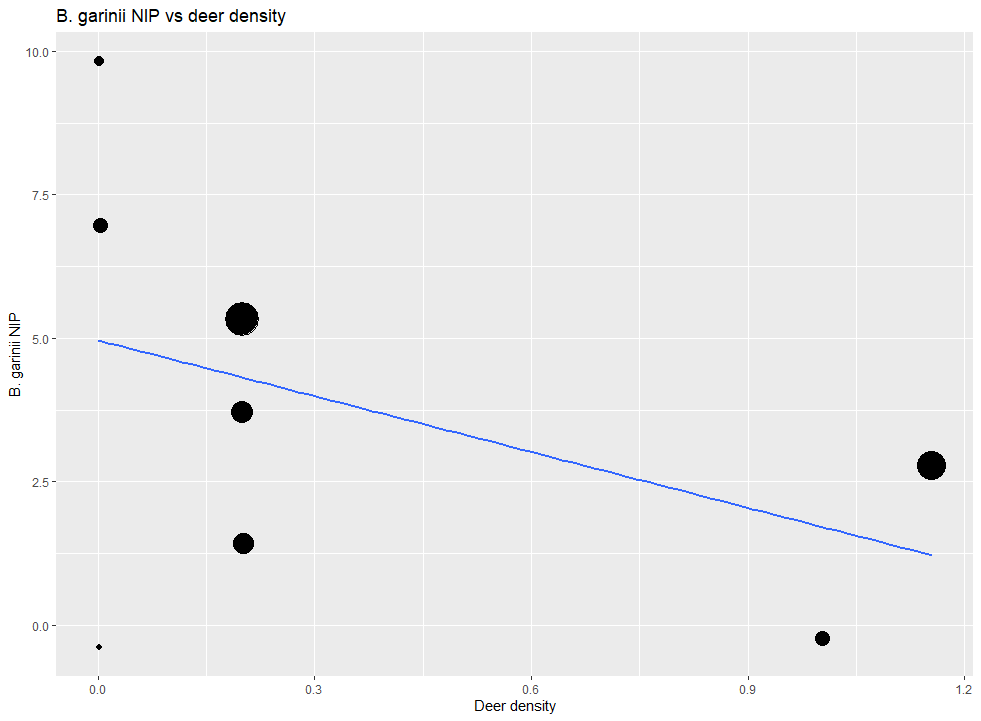  p = 0.061 | 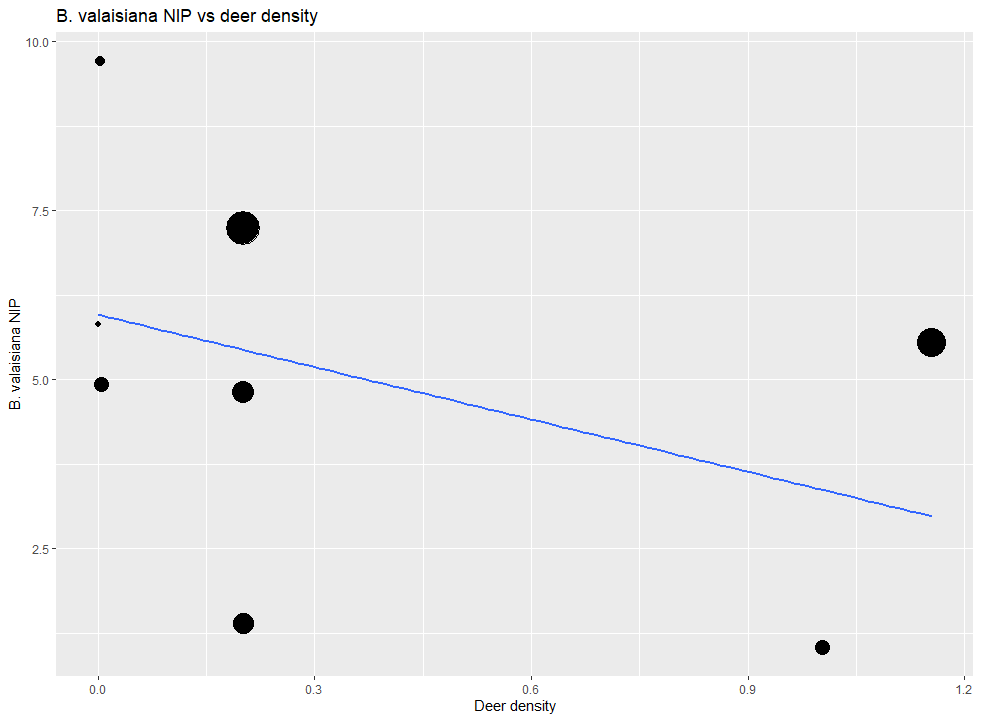  p = 0.39 |
| --- | --- |
| 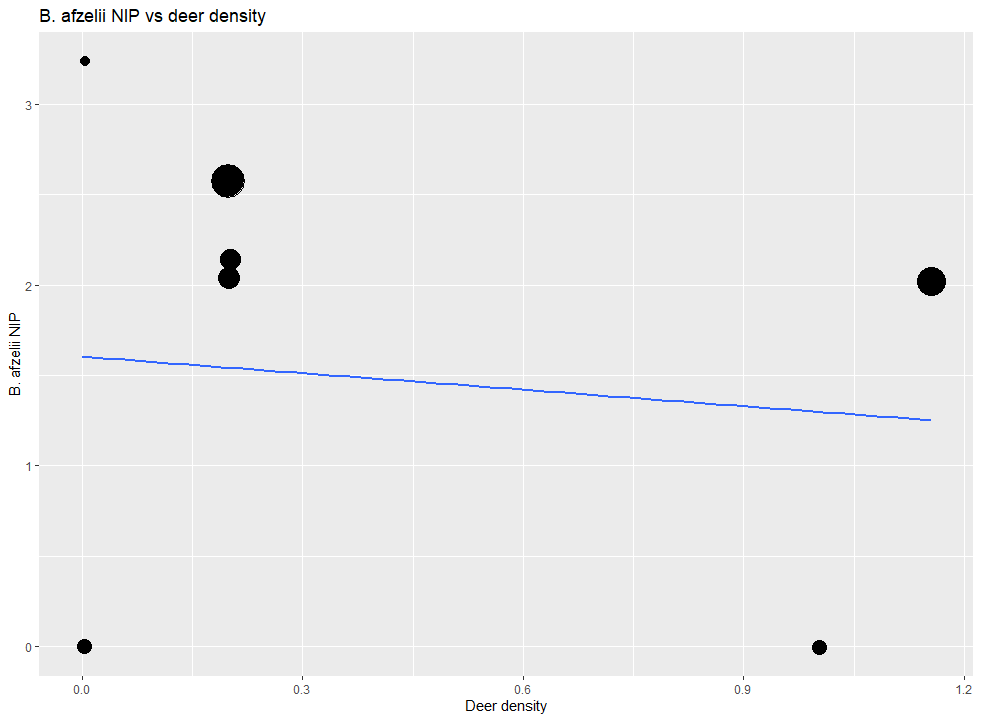  p = 0.7176 | 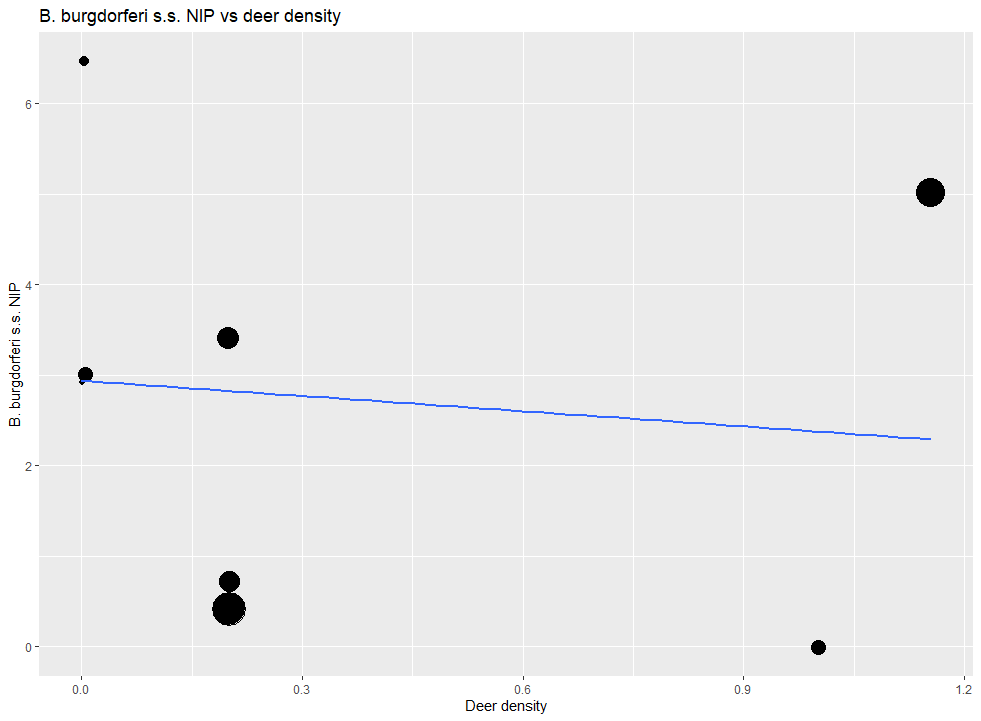  p = 0.9555 |

Figure 13 Bubble plots and p-values for genospecies-specific NIPs by deer density in Ireland. The lack of significant -p-values in this figure demonstrates that there was no significant relationship between NIP and deer density for any of the four strains.

## NIP/DIN by location in Ireland

The figures in this section display the data that pertain to section 2.4.4 of the manuscript. Meta-analyses subgrouped by location in Ireland were conducted to assess the possibility of over-representation of certain areas in Ireland.


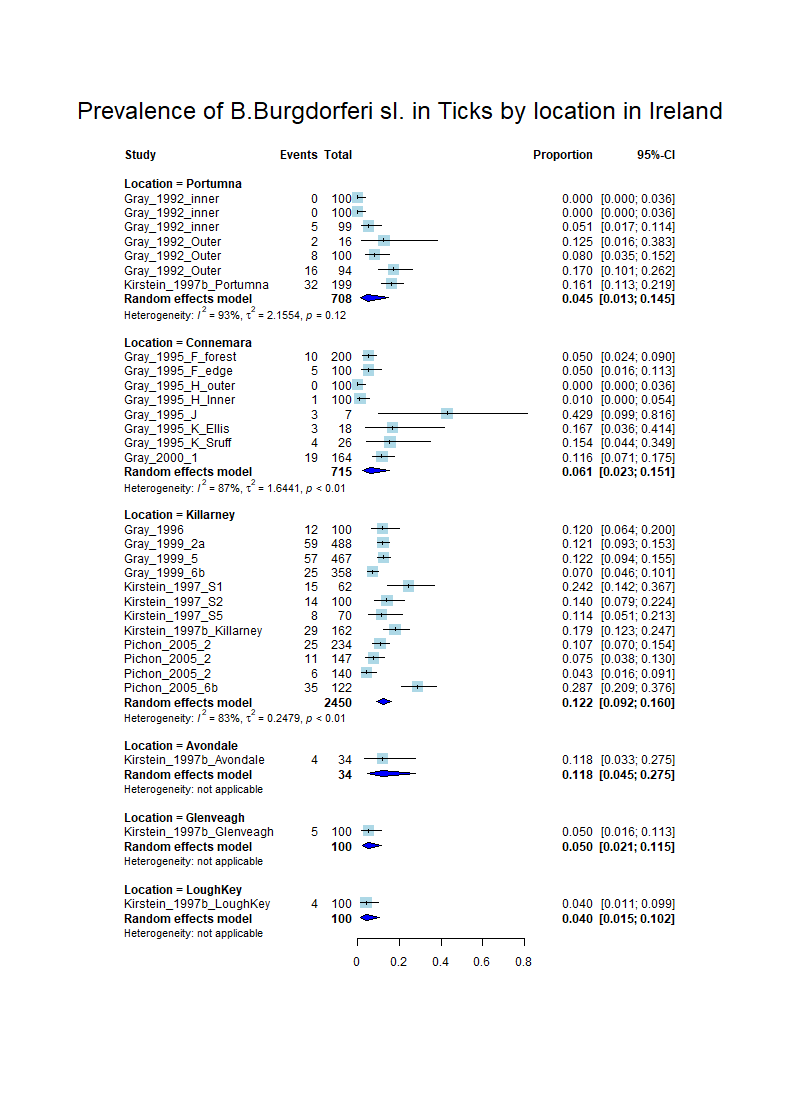


**Prevalence of *B. burgdorferi* s.l*.* in ticks by location in Ireland**

Figure 14 B. burgdorferi s.l. NIP subgrouped by location of woodland site in Ireland. The confidence intervals for the weighted average NIP for all locations overlap.


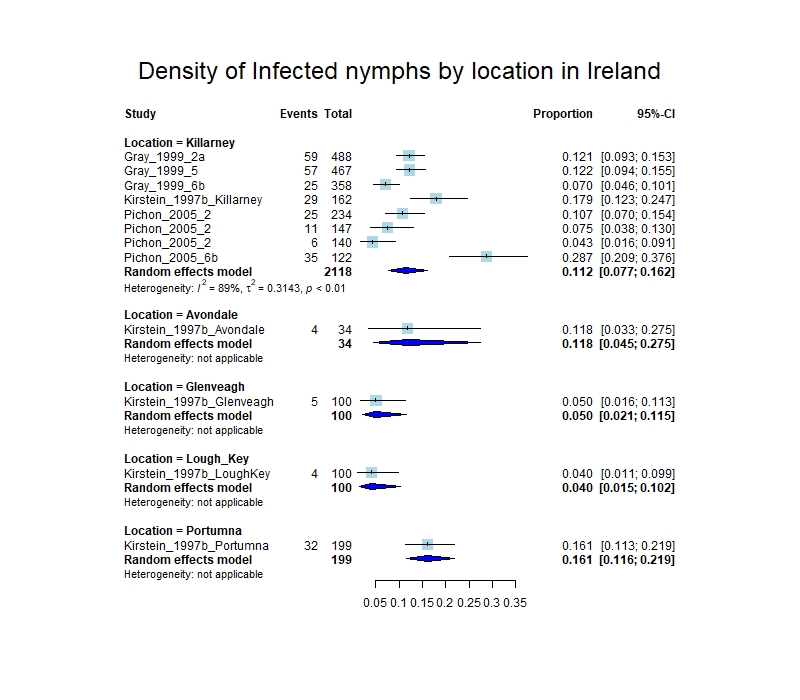


Figure 15 B. burgdorferi s.l. DIN subgrouped by location of woodland site in Ireland. The confidence intervals for the weighted average NIP for all locations overlap, apart from Portumna, which does not overlap with Lough Key or Glenveagh.

Figure 14 shows that the confidence intervals for NIP for all sites subgrouped by location overlap. This indicates that though regions such as Portumna, Killarney, and Connemara were well represented, they would not have unduly influenced the outcome of the overall meta-analysis for NIP in Ireland.

Figure 15 shows that the confidence intervals for DIN for most sites subgrouped by location overlap. The exception here is Portumna, which does not overlap with Lough Key or Glenveagh. However, as there is only one eligible site for each of these it cannot be stated that they are over-represented. The location that is most represented, Killarney, has a confidence interval which overlaps with all other locations in this analysis, indicating that this location would not have unduly influenced the outcome of the overall meta-analysis for DIN in Ireland.
